# Causal relationship between 731 immune cells and the risk of diabetic nephropathy: a two‑sample bidirectional Mendelian randomization study

**DOI:** 10.1080/0886022X.2024.2387208

**Published:** 2024-08-01

**Authors:** Siyuan Song, Yuqing Sun, Jiangyi Yu

**Affiliations:** Department of Endocrinology, Jiangsu Province Hospital of Chinese Medicine Affiliated to Nanjing University of Chinese Medicine, Nanjing University of Chinese Medicine, Jiangsu, Nanjing, P.R. China

**Keywords:** Mendelian randomization analysis, bidirectional, immune cells, diabetic nephropathy, myeloid dendritic cell

## Abstract

**Objective:**

Previous observational studies have indicated associations between various immune cells and diabetic nephropathy (DN). However, the causality remains unclear. We aimed to further evaluate the causal association between immune cells and DN using bidirectional two-sample Mendelian randomization (MR) analysis.

**Method:**

The DN data were retrieved from the IEU OpenGWAS Project database, while the data for 731 immune cells were sourced from GWAS summary statistics by Orru ` et al. The investigation into the causal relationship between immune cells and DN employed the inverse variance weighted (IVW), weighted median (WME), and MR-Egger methods. The stability and reliability of the findings underwent evaluation through Cochran’s Q test, MR-Egger intercept’s *P*-value, MR-PRESSO, and Leave-One-Out (LOO) method.

**Result:**

The IVW estimates suggested a positive causal effect of CD25 on IgD-CD38dim B cell, CD25 on naive-mature B cell, CD127 on granulocyte, SSC-A on HLA DR + Natural Killer, HLA DR on plasmacytoid Dendritic Cell, and HLA DR on Dendritic Cell on DN. Conversely, the abundance of Myeloid Dendritic Cell, CD62L- Dendritic Cell %Dendritic Cell, CD86+ myeloid Dendritic Cell %Dendritic Cell, CD14- CD16-, CX3CR1 on CD14- CD16-, and SSC-A on CD4+ T cell had negative causal effects on DN. However, after correcting the *P* value for significant causality results using the FDR method, it was concluded that only Myeloid Dendritic Cells had a causal relationship with DN (FDR-*p* = 0.041), while the other immune cells showed no significant association with DN, so their relationship was suggestive. The results were stable with no observed horizontal pleiotropy and heterogeneity. Reverse MR analysis indicated no causal relationship between DN and the increased risk of positively identified immune cells.

**Conclusion:**

This study provides an initial insight into the genetic perspective of the causal relationship between immune cells and DN. It establishes a crucial theoretical foundation for future endeavors in precision medicine and individualized treatment.

## 1. Introduction

Diabetic nephropathy (DN) commonly occurs as a complication of type 2 diabetes mellitus (T2DM). Currently, there is a prevailing belief that the interplay among inflammatory factors, endocrine functions, the immune system, oxidative stress, and abnormal fat metabolism contributes to deviations in cellular structure, islet resistance, and microvascular complications [1]. Despite numerous studies confirming that renin-angiotensin system inhibitors (RASi), sodium-glucose linked transporter-2 inhibitors (SGLT-2i), and novel mineralocorticoid receptor antagonists (MRA) can significantly reduce renal composite endpoints and are recommended by guidelines at various levels and widely used in clinical practice, the incidence of DN and its progression to end-stage kidney disease (ESKD) continue to rise [2]. Thus, it is imperative to carry out comprehensive research on the underlying mechanisms and potential causative factors to devise effective treatments.

Increasing evidence indicates that damaged kidneys are enriched with immune cells such as T lymphocytes, B lymphocytes, and macrophages within the glomeruli and interstitium. These cells activate both innate and adaptive immune responses in the kidney, driving structural remodeling and interstitial fibrosis, which ultimately leads to renal function decline [3,4]. Consequently, immune cells play a pivotal role in the pathogenesis and progression of DN [5]. However, current research on the pathogenesis of DN predominantly relies on observational studies and fundamental experimental research [6,7], which have inherent limitations in inferring causality. Although these studies can reveal specific immune cell alterations associated with DN, they fail to establish whether these changes are causally related to DN. Moreover, the causal relationships between various immune cells and the progression of DN remain inadequately explored. Therefore, additional evidence is needed to substantiate the causal association between immune cells and DN.

**Figure 1.**
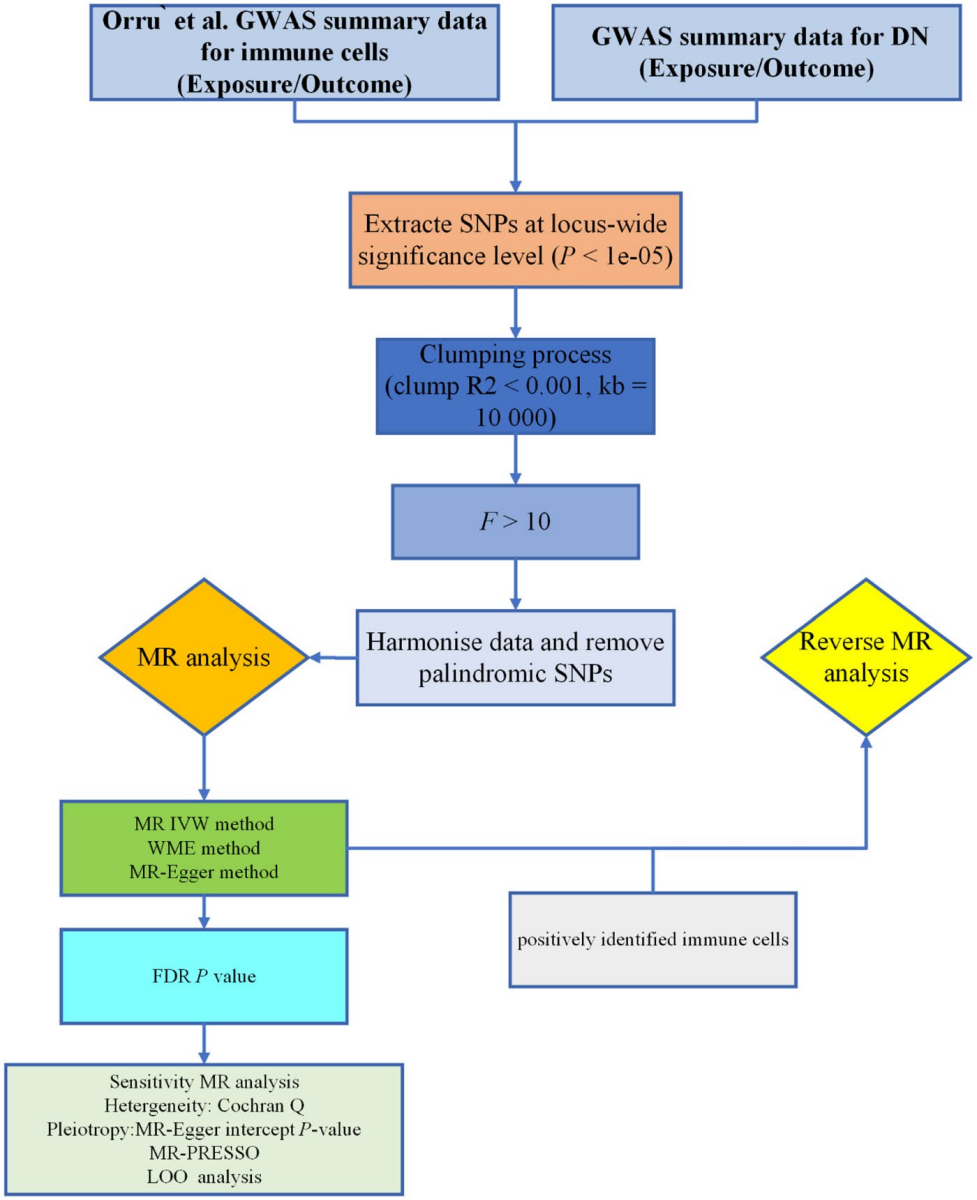
The protocol of our study procedure. GWAS: Genome-wide association study; DN: diabetic nephropathy; IVW: Inverse variance weighted; SNPs: Single nucleotide polymorphisms; LOO: Leave-one-out; MR: Mendelian randomization; WME: Weighted median.

Mendelian randomization (MR) is a genetic variable analysis method that follows Mendel’s laws of inheritance [8]. It uses single-nucleotide polymorphisms (SNPs) as instrumental variables (IVs) to infer causal relationships between observed modifiable exposure factors and clinically relevant outcomes [9]. Since alleles are randomly separated during meiosis, MR analysis can reduce bias caused by confounding factors. Moreover, because genetic variations occur before the onset of diseases, the temporal sequence between them cannot be reversed, allowing MR analysis to avoid the interference of reverse causality [10]. Therefore, genetic variations closely related to exposure factors are now often used as IVs to infer causal relationships between exposure factors and study outcomes. In this study, a two-sample bidirectional MR analysis was employed to investigate the causal relationship between immune cells and DN, offering genetic support for their association. The study protocol is outlined in Figure 1.

## 2. Material and method

### 2.1. Data sources

We selected the peripheral blood immunophenotype for analyzing GWAS data, and the summary statistics of 731 immune traits were publicly provided in the GWAS catalog database. The GWAS analysis is based on the European population, including 3,757 European adults. After adjusting for gender and age, approximately 220,000 single-nucleotide polymorphisms (SNPs) were tested [11]. Among the 731 immune cells, 118 represented absolute cell counts, 389 reflected the median fluorescence intensity, 32 were morphological parameters, and 192 were relative cell counts. The DN data (ebi-a-GCST90018832) originated from the IEU OpenGWAS Project website (gwas.mrcieu.ac.uk), which comprises 452,280 samples, with 24,190,738 SNPs from East Asian and European [12]. The DN dataset finn-b-DM_NEPHROPATHY was used for GWAS dataset validation, which comprises 210,463 samples and 16,380,453 SNPs from the European population. As this study relies on public data, no additional ethical approval or consent is required.

### 2.2. Method

**Figure 2.**
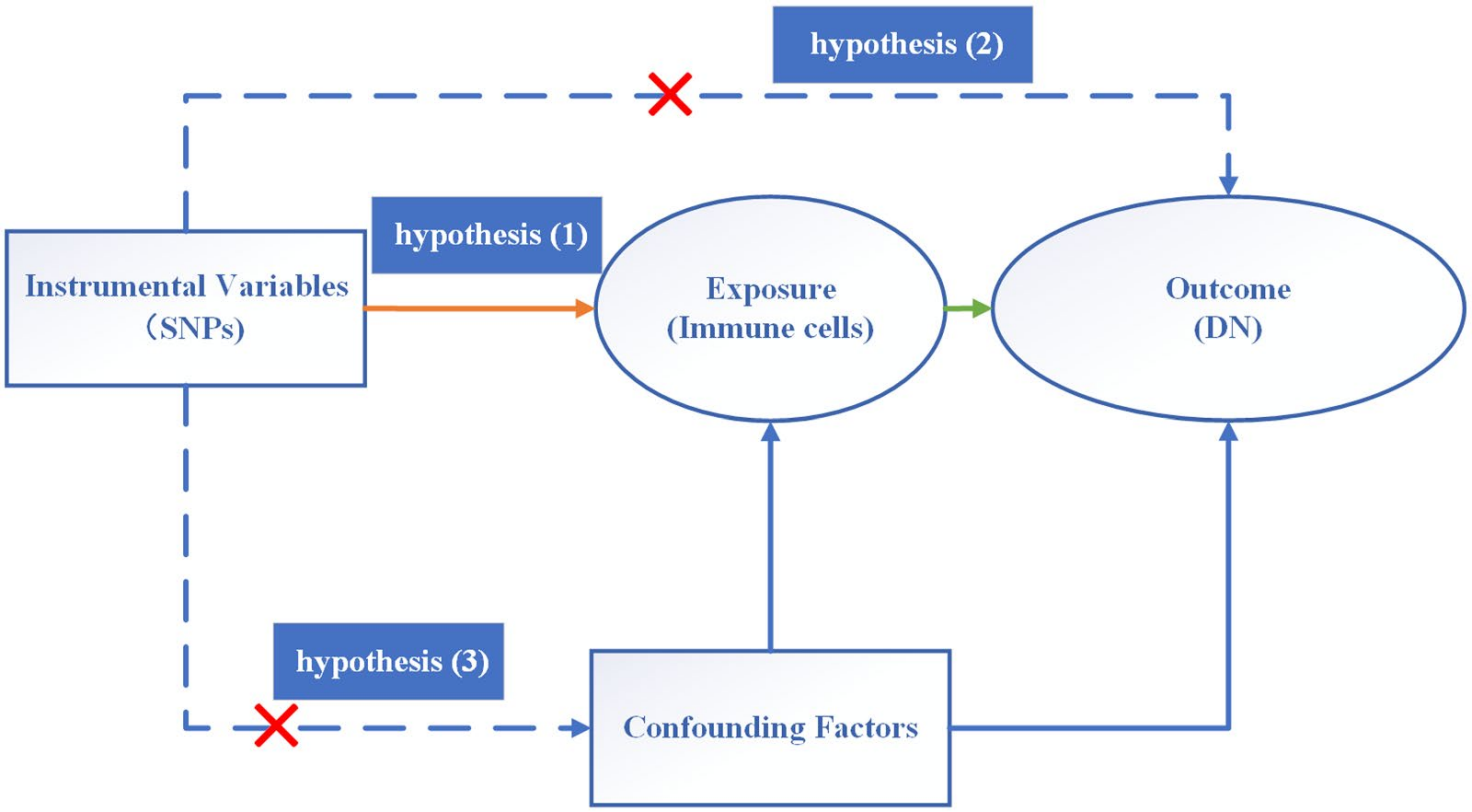
The hub hypothesis of the MR analysis. SNPs: Single-nucleotide polymorphisms; DN: diabetic nephropathy.

MR analysis utilized SNPs as IVs to examine the causal relationship between exposure and outcome. The selection of IVs in this study should adhere to three assumptions [13]: (1) There is a strong correlation between IVs and exposure (immune cells); (2) IVs are not directly related to the outcome (DN); (3) There is no correlation between IVs and confounding factors. Firstly, the following conditions should be met: genome-wide significance level (*P* < 1e-05) and linkage disequilibrium threshold (kb = 10000, R2 < 0.001) [14,15]. Secondly, the PhenoScanner database [16] was utilized to further validate whether the identified SNP loci exhibited an association with other potential confounding factors (BMI, blood pressure, blood lipids, heart disease). The study excluded the following genetic variants based on BMI: rs10406080, rs754388, rs17437411, rs60699901, and rs2267373. Additionally, rs10919543 and rs6429147 were disregarded due to their association with blood pressure. Similarly, rs1326634 and rs7819412 were omitted because of their relationship to blood lipids. Finally, rs621559, rs2330634, and rs283227 were excluded from the analysis due to their connection with heart disease. Thirdly, to assess the susceptibility of the included SNPs to weak IV bias, F statistics were employed, with a threshold set at *F* > 10 (calculated using the formula F = β^2/SE^2, where β represents the effect on the exposure and SE represents the standard error). SNPs with *F* < 10 were considered susceptible to weak IV bias and were consequently excluded to prevent their influence on the results [17]. The hub hypothesis of the MR analysis is depicted in Figure 2.

### 2.3. Statistical analysis

#### 2.3.1. MR analysis

To assess the causal relationship between immune cells and DN, primarily the IVW method, WME method, and MR-Egger regression method were utilized. The IVW method is the main analytical method used to determine causal relationships. It primarily weights the causal effects of different genetic variants on traits and calculates the weighted effects, thereby clarifying the causal effects of genes on traits. This method has the advantages of optimizing weight allocation and reducing the influence of confounding factors [18]. The WME method assigns different weights to genetic variants and reduces the impact of extreme genetic variants on causal effects, enhancing the stability of causal relationship estimates [19]. The MR-Egger method includes tests for directional pleiotropy, causal effect testing, and calculation of causal effect estimates, allowing the evaluation of the multiple impacts of genetic variants on traits [20]. Because the IVW method combines effect estimates from individual genetic variants by weighting them inversely to their variances. By assigning higher weight to more precise estimates, IVW enhances the reliability of the overall causal effect estimate [21–24]. Therefore, this study adopts the IVW method as the main analytical method, with MR-Egger and WME methods as supplementary methods for causal inference. The *FDR* method was employed to control the false discovery rate when adjusting the *P* value. The FDR-adjusted *p* < 0.05 was deemed indicative of a significant causal relationship between the exposure and the outcome. The FDR-adjusted *p* < 0.2 suggested a potential causal relationship between the exposure and the outcome [25].

#### 2.3.2. Sensitivity analysis

To assess the robustness of the causal effect of immune cells on DN, we conducted a series of sensitivity analyses. Cochran’s Q analysis was employed to calculate differences among IVs, with heterogeneity considered present when *p* < 0.05 [26]. Based on the presence of heterogeneity, the choice between testing a random effect model or a fixed effect model was determined. The funnel plot was used to detect heterogeneity, and a symmetrical distribution of SNPs indicated no heterogeneity in the results [27]. Horizontal pleiotropy was assessed through the intercept term of the MR-Egger test and MR-PRESSO analysis. MR-PRESSO serves as the principal approach for assessing horizontal pleiotropy, with strict requirements for application. Effective utilization demands that a minimum of 50% of the IVs demonstrate efficacy. When the *P*-value of the MR-Egger intercept surpasses 0.05, horizontal pleiotropy lacks statistical significance, affirming the validity of the exclusionary hypothesis [28]. LOO analysis was employed to test data stability, determining whether there were SNPs that exerted a strong influence when a single SNP was removed [29]. The effect size in this study is expressed as odds ratio (OR) and 95% confidence interval (CI).

#### 2.3.3. Bidirectional MR analysis

We performed a two-sample bidirectional MR analysis to investigate the reciprocal causal relationship between DN (exposure) and immune cells that were positively identified (outcome). The steps for bidirectional MR analysis mirror those of the standard MR analysis.

#### 2.3.4. Functional enrichment analysis by the selected SNPs

For a better understanding of the biological process of the selected SNPs, appraisal of the functional annotations of the genetic variants regarded as IVs in prior MR analysis was carried out in FUMAGWAS tool. Afterward, the functional enrichment analysis was searched according to the genes by the DAVID database [30].

#### 2.3.5. Statistical software

The MR analyses were conducted using R (version 4.3.1), the TwoSample MR package, and the MR-PRESSO package.

## 3. Results

### 3.1. Instrumental variable selection

**Figure 3.**
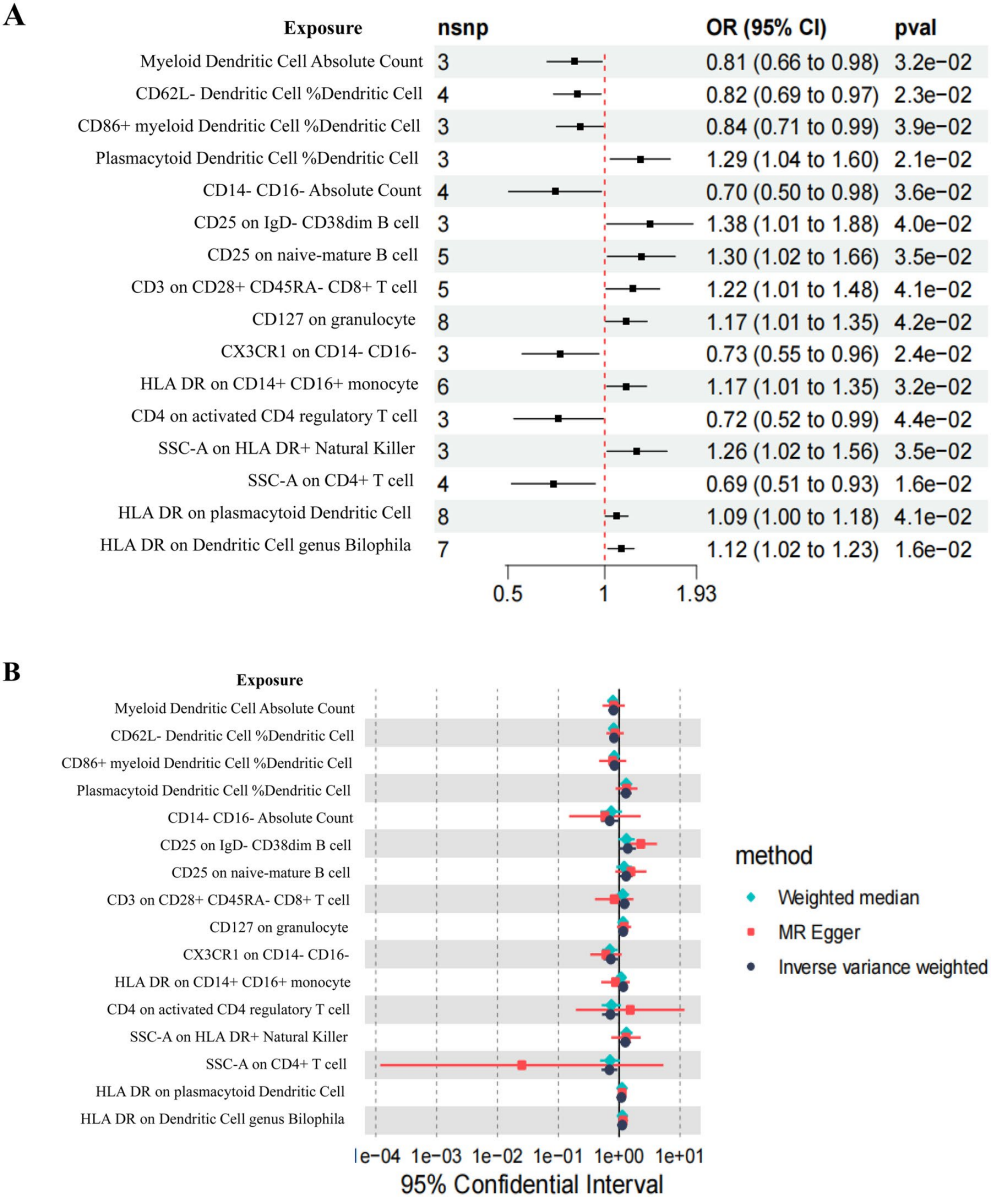
MR estimates of the causal effects of immune cells on DN.(A) Forest plot of IVs selection.(B) Forest plot of MR results.The green diamond signifies the WME method, the red square corresponds to the MR-Egger method, and the grey circle represents the IVW method.

Following the IVs screening criteria, we obtained 73 SNPs from 731 immune cells, and each SNP’s F-statistics exceeded 10 (Supplementary Table 1), indicating resilience to the influence of weak IVs bias. The IVW method was primarily employed in this study, and immune cells were analyzed by MR, considering *p* < 0.05 as the threshold (Figure 3(A)).

### 3.2. MR analysis

IVW analysis revealed that the CD25 on IgD- CD38dim B cell (OR = 1.382, 95%CI 1.014–1.883, *p* = 0.040), CD25 on naive-mature B cell (OR= 1.299, 95%CI 1.018–1.658, *p* = 0.035), CD127 on granulocyte (OR = 1.167, 95%CI 1.006–1.355, *p* = 0.042), SSC-A on HLA DR + Natural Killer (OR = 1.260, 95%CI 1.016–1.563, *p* = 0.035), HLA DR on plasmacytoid Dendritic Cell (OR = 1.089, 95%CI 1.003–1.182, *p* = 0.041), Plasmacytoid Dendritic Cell %Dendritic Cell (OR = 1.288, 95%CI 1.030–1.600, *p* = 0.020), and HLA DR on Dendritic Cell (OR = 1.123, 95%CI 1.022–1.234, *p* = 0.016) had positive effects on DN, whereas the Myeloid Dendritic Cell Absolute Count (OR = 0.806, 95%CI 0.661–0.982, *p* = 0.032), CD62L- Dendritic Cell %Dendritic Cell (OR = 0.821, 95%CI 0.693–0.974, *p* = 0.023), CD86+ myeloid Dendritic Cell %Dendritic Cell (OR = 0.837, 95%CI 0.707–0.991, *p* = 0.039), CD14- CD16- Absolute Count (OR = 0.700, 95%CI 0.502–0.9773, *p* = 0.036), CX3CR1 on CD14- CD16- (OR = 0.728, 95%CI 0.553–0.959, *p* = 0.024), and SSC-A on CD4+ T cell (OR = 0.692, 95%CI 0.512–0.935, *p* = 0.016) exhibited negative effects on DN (Figure 3(B)). The forest plot of single SNP MR results is presented in Supplementary Figure 1. The WME analysis corroborated the aforementioned conclusions. However, the results for CD3 on CD28+ CD45RA- CD8+ T cell, HLA DR on CD14+ CD16+ monocyte, CD4 on activated CD4 regulatory T cell, and DN showed total effect values in the opposite direction for MR-Egger and IVW, leading to their exclusion (Figure 4(A–P)).

Figure 4.Scatter plots of SNP analysis.(A) Myeloid Dendritic Cell Absolute Count;(B) CD62L- Dendritic Cell %Dendritic Cell;(C) CD86+ myeloid Dendritic Cell %Dendritic Cell;(D) Plasmacytoid Dendritic Cell %Dendritic Cell;(E)CD14- CD16- Absolute Count;(F) CD25 on IgD- CD38dim B cell;(G) CD25 on naive-mature B cell(H) CD3 on CD28+ CD45RA- CD8+ T cell;(I) CD127 on granulocyte;(J)CX3CR1 on CD14- CD16-;(K) HLA DR on CD14+ CD16+ monocyte;(L) CD4 on activated CD4 regulatory T cell;(M) SSC-A on HLA DR + Natural Killer;(N) SSC-A on CD4+ T cell;(O) HLA DR on plasmacytoid Dendritic Cell;(P) HLA DR on Dendritic Cell genus Bilophila.The X-axis denotes the impact of SNP on the immune cell, the Y-axis signifies the effect of SNP on DN, the black dot signifies a single SNP, the line segment represents the 95% CI, and the slope of the straight line indicates the causal estimation of the MR method. The light blue line corresponds to the IVW method, the blue line corresponds to MR-Egger, and the green line corresponds to the WME method.

**Figure:**
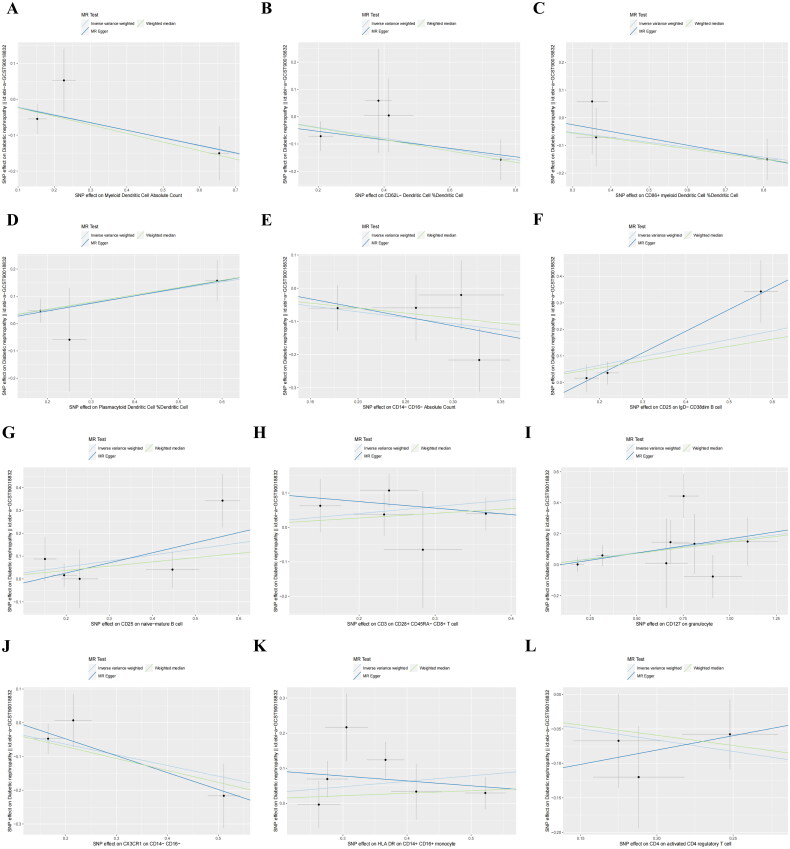

**Figure:**
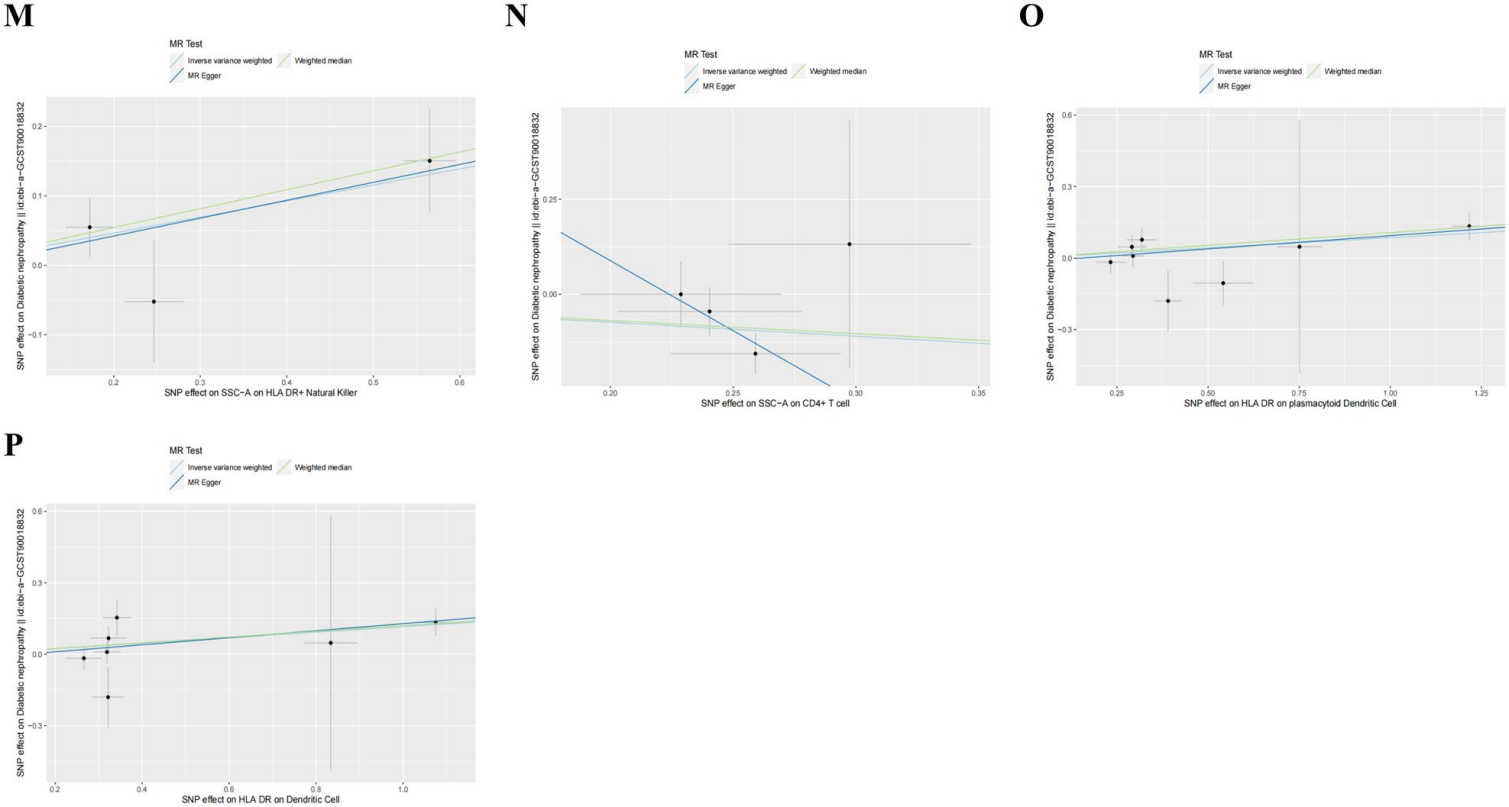

After correcting the *P* value for significant causality results using the FDR method, it was concluded that only Myeloid Dendritic Cell Absolute Count had a causal relationship with DN (*FDR-p* = 0.041), while the other 15 immune cells showed no significant association with DN (Supplementary Table 2), so their relationship was suggestive [31,32].

### 3.3. Sensitivity analysis

Cochran’s Q test indicated no heterogeneity in the causal relationship between immune cells and DN (*p* > 0.05), prompting the use of a fixed-effect model for analysis. The intercept tests of MR-Egger regression and MR-PRESSO methods revealed no horizontal pleiotropy in the significant causal relationship (Supplementary Table 3). Funnel plots suggested that potential interference factors were unlikely to impact causality (Supplementary Figure 2). LOO analysis demonstrated the stability of significant causality after sequentially removing SNPs (Figure 5(A–M)).

Figure 5.Forest Plots of LOO sensitivity analysis.(A) Myeloid Dendritic Cell Absolute Count;(B) CD62L- Dendritic Cell %Dendritic Cell;(C) CD86+ myeloid Dendritic Cell %Dendritic Cell;(D) Plasmacytoid Dendritic Cell %Dendritic Cell;(E) CD14- CD16- Absolute Count;(F) CD25 on IgD- CD38dim B cell;(G) CD25 on naive-mature B Cell;(H) CD127 on granulocyte;(I) CX3CR1 on CD14- CD16-;(J) SSC-A on HLA DR + Natural Killer;(K) SSC-A on CD4+ T cell;(L) HLA DR on plasmacytoid Dendritic Cell;(M) HLA DR on Dendritic Cell.The black dot signifies the DN with increased standard deviation (SD) in the immune cell, generated by using each SNP as a separate instrumental variable. The red dot represents the causal estimation of all SNP combinations by different MR methods. The horizontal line segment signifies the 95% CI. The IVW causal estimate and the impact of removing a single variant on the overall estimate (red horizontal line) were visualized.

**Figure:**
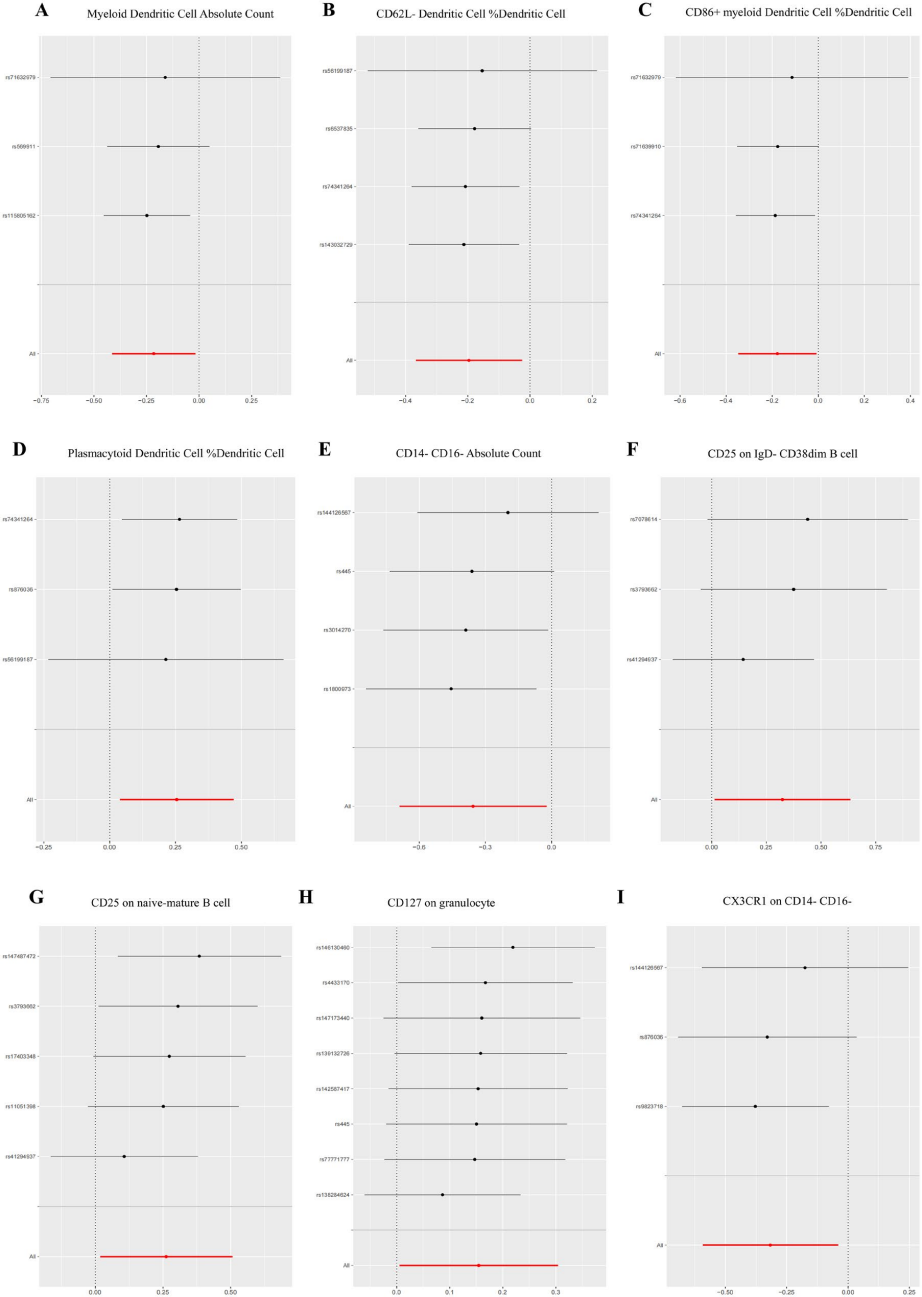

**Figure:**
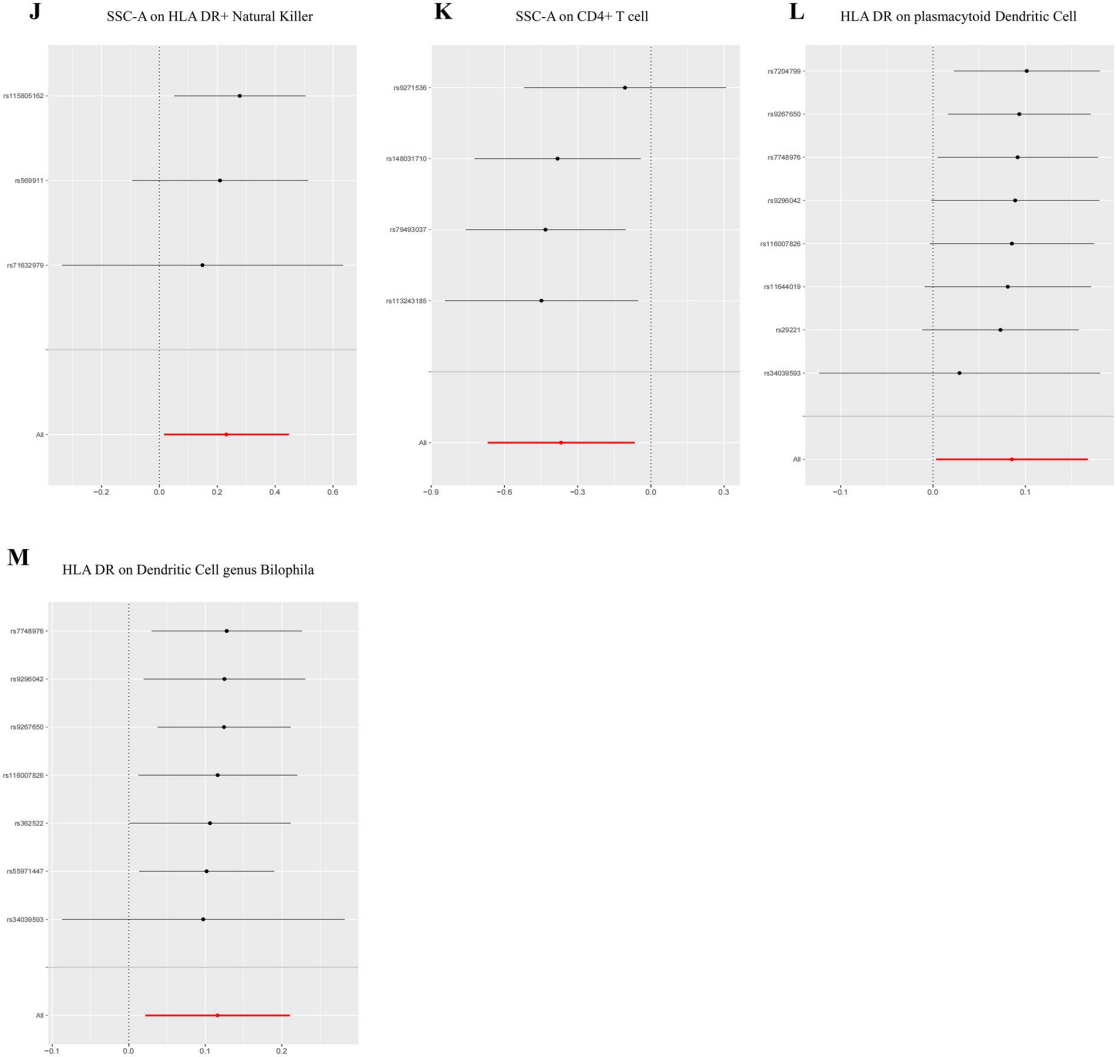

### 3.4. Bidirectional MR analysis

Reverse MR analysis results indicated no causal relationship between DN and the heightened risk of positively identified immune cells (Figure 6(A–M)).

Figure 6.Forest plot of the bidirectional MR analysis of the positively identified 15 immune cells.(A) Myeloid Dendritic Cell Absolute Count;(B) CD62L- Dendritic Cell %Dendritic Cell;(C) CD86+ myeloid Dendritic Cell %Dendritic Cell;(D) Plasmacytoid Dendritic Cell %Dendritic Cell;(E) CD14- CD16- Absolute Count;(F) CD25 on IgD- CD38dim B cell;(G) CD25 on naive-mature B cell(H) CD127 on granulocyte;(I) CX3CR1 on CD14- CD16-;(J) SSC-A on HLA DR + Natural Killer;(K) SSC-A on CD4+ T cell;(L) HLA DR on plasmacytoid Dendritic Cell;(M) HLA DR on Dendritic Cell genus Bilophila.

**Figure:**
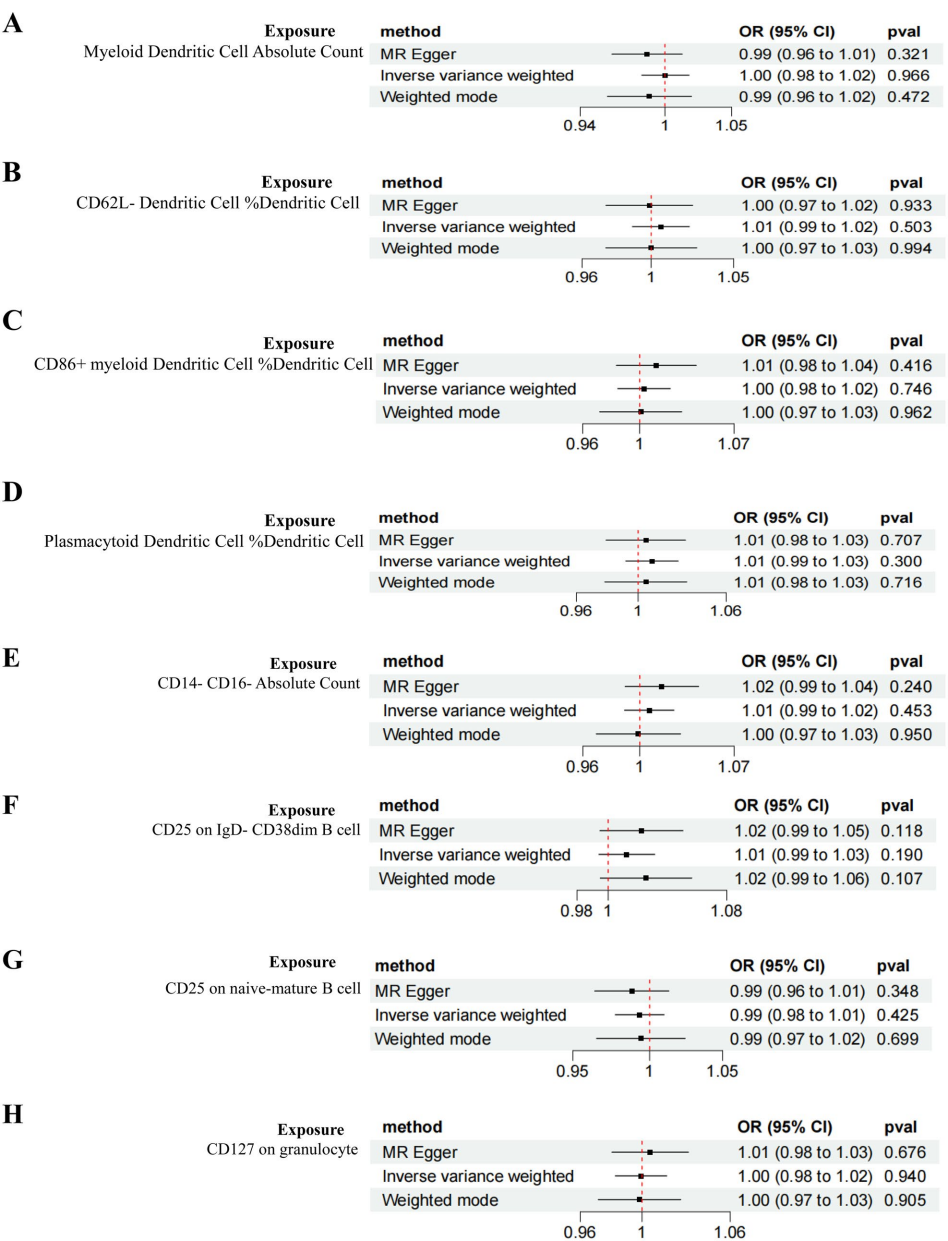

**Figure:**
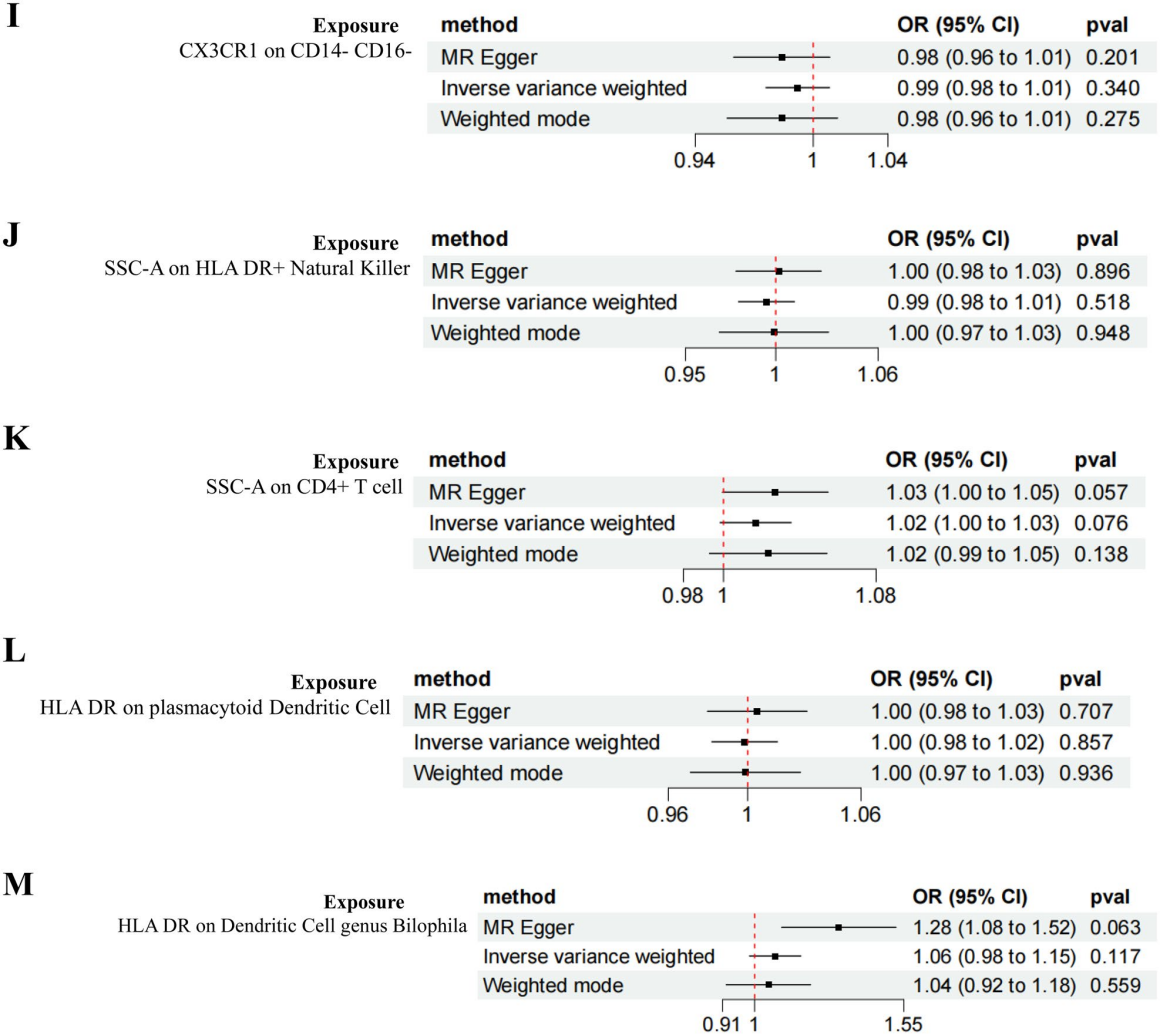

### 3.5. Dataset validation

**Figure 7.**
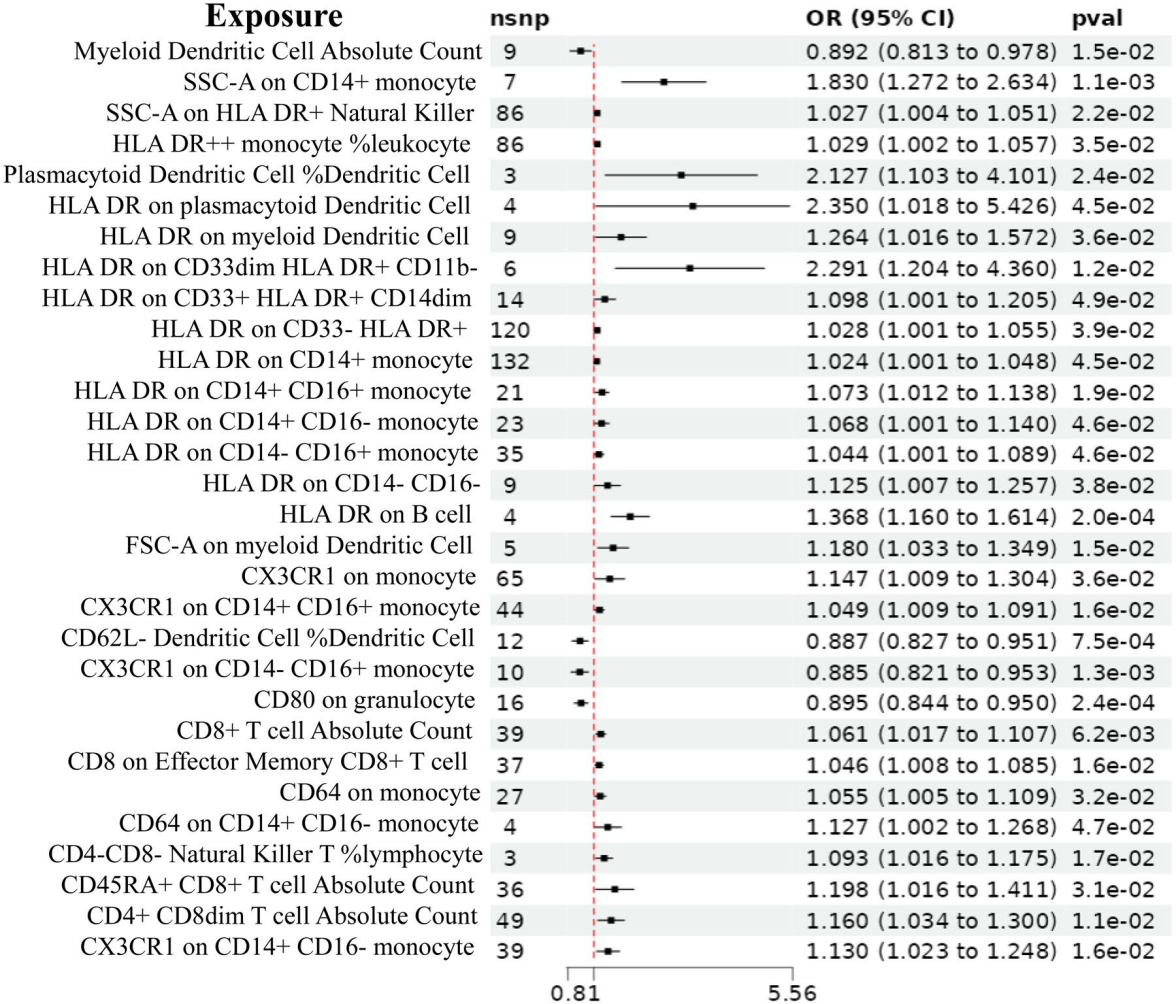
MR estimates of the causal effects of immune cells on DN in the validation GWAS data.

In the validation GWAS data finn-b-DM_NEPHROPATHY, the IVW analysis revealed that the HLA DR on plasmacytoid Dendritic Cell (OR = 2.350, 95%CI 1.018–5.426, *p* = 4.5e-02), Plasmacytoid Dendritic Cell %Dendritic Cell (OR = 1.029, 95%CI 1.002–1.057, *p* = 3.5e-02), and SSC-A on CD14+ monocyte (OR = 1.830, 95%CI 1.272–2.634, *p* = 1.1e-03) had positive effects on DN, whereas the Myeloid Dendritic Cell (OR = 0.082, 95%CI 0.813–0.978, *p* = 1.5e-02) exhibited negative effects on DN (Figure 7), which further verified the reliability of the findings in this study.

### 3.6. Functional enrichment analysis by the selected SNPs

**Figure 8.**
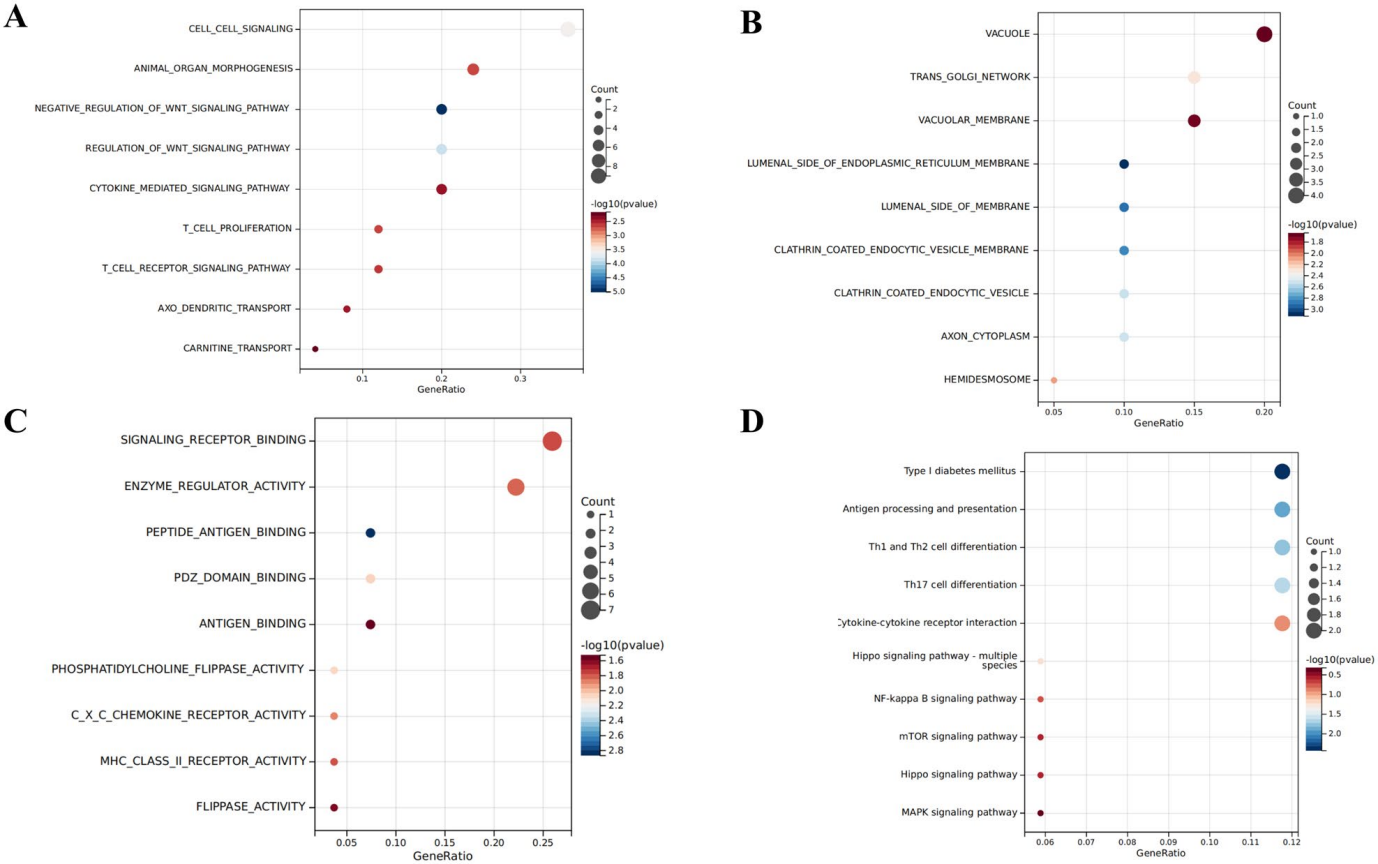
Bubble diagram for functional enrichment analysis.(A) Biological functions of genes;(B) Cell components of genes;(C) Molecular functions of genes;(D) KEGG of genes. The bubble size represents the number of enriched genes, and the bubble color difference represents the significant magnitude of target gene enrichment.

We finally got 62 genes mapped from the selected SNPs, functional enrichment analysis showed that the biological functions mainly involved cell-cell signaling, T-cell proliferation, and T-cell receptor signaling pathway cell proliferation. Cell components mainly included the axon cytoplasm, hemidesmosome, and vacuole. Molecular functions mainly included MHC class II receptor activity, flippase activity, and antigen binding. Kyoto Encyclopedia of Genes and Genomes (KEGG) functional enrichment analysis showed that the genes were mainly enriched in antigen processing and presentation, Th1 and Th2 cell differentiation, Th17 cell differentiation, Hippo signaling pathway-multiple species, and NF-kappa B signaling pathway (Figure 8), suggesting that the identified immune cells may treat DN through these pathways.

## 4. Discussion

Immune system plays an important role in the pathogenesis of DN, and it has been paid more and more attention by basic and clinical research. However, at present, the genetic relationship between them is not clear, so this study uses a two-sample bidirectional MR method to explore the causal relationship between 731 kinds of immune cells and DN. In this study, after correcting the *P* value for significant causality results using the FDR method, it was concluded that only Myeloid Dendritic Cell had a causal relationship with DN, while the other 15 immune cells showed no significant association with DN, so their relationship was suggestive. Further sensitivity analysis results showed that the above results are consistent and reliable. However, the results of reverse MR analysis showed that there was no causal relationship between DN and the increased risk of positively identified immune cells.

Studies have shown that DN is closely related to immune cells, and the structural composition of immune cells can affect DN [33]. The results of renal biopsy in DN patients also showed that immune inflammatory cells existed in the glomeruli and interstitium of DN and were closely related to glomerulosclerosis, tubular atrophy, and interstitial fibrosis [34]. Many studies have shown that CD25 on immune cells is related to various inflammatory diseases [35–37]. HLA-DR molecules on the surface of immune cells have anti-inflammatory effects [38]. CD4+ T cells and CD8+ T cells in the renal interstitium of patients with T2DM are significantly increased, and the number of CD4+ T cells is positively correlated with the amount of proteinuria [39]. In addition, a clinical study involving 89 patients with type 1 diabetes mellitus (T1DM) showed that the accumulation of T cells in the organs adjacent to glomerulus would aggravate diabetes, and it was related to the glomerular filtration area and urinary protein excretion rate [40]. Bending [41] found that the level of circulating T lymphocytes in the proteinuria group was significantly higher than that in the nonproteinuria group in the patients with T1DM. Lampropoulou [42] showed that in the patients with DN, circulating T lymphocyte activation markers increased with the severity of proteinuria. The above studies suggest that T cells are involved in the occurrence and development of DN and are related to the disease progression. Previous studies have shown that there is IgG + B-cell infiltration in the glomerulus of diabetic NOD mice [43]. In human diabetic kidneys, the number of B cells increased significantly, indicating that B cells may participate in the progress of DN. In addition, a study on B cells in the peripheral blood of DN patients showed that the number of CD38+ CD19+ B cells in DN patients was positively correlated with the 24-h urine protein concentration, and negatively correlated with glomerular filtration rate, suggesting that the higher level of B cells was related to the deterioration of DN [44]. The ratio value of neutrophilic granulocyte to lymphocytic granulocyte (NLR) is the index reflecting the degree of inflammation, it has the clinical value of early detection of DN [45]. Monocytes include macrophages and dendritic cells. In a high glucose environment, after the mononuclear macrophage cell system is activated, macrophages infiltrate into the kidney and promote the release of cytokines. The release of cytokines and the infiltration of mononuclear macrophages eventually lead to inflammatory damage to the kidney [46].

B cells promote the development of DN mainly by producing antibodies and forming immune complexes that are deposited in the kidneys [47,48]. The effects of dendritic cells on DN are less well studied, but the basic viewpoints focus on the ability of dendritic cells to present antigens, activate T lymphocytes, and mediate immune inflammation associated with diabetic complications [49,50]. Dendritic cells originating from the myeloid lineage are also known as conventional dendritic cells (cDCs). Based on different surface molecules, cDCs can be further divided into cDC1 and cDC2 [51]. Human cDC1 primarily expresses CD141 on its surface [52]. cDC1 can recognize intracellular pathogens and trigger a CD8+ T-cell response. Additionally, they enhance the immune effects of type 1 helper T cells (Th1) and natural killer (NK) cells through cytokines such as IL-12 [53]. cDC2 is the most predominant dendritic cell type in human blood, tissues, and lymphoid organs. Human cDC2 primarily expresses CD1c on its surface and has a stronger ability to secrete IL-12. In specific environments, cDC2 can secrete IL-8, IL-6, and other cytokines. cDC2 can stimulate the activation of Th1, Th2, and Th17 cells, thereby possessing a broad range of immune response capabilities [54]. NK cells are the main source of γ -interferon and γ -interferon is an important proinflammatory factor [55]. Mature myeloid dendritic cells are effective stimulators of T cell immunity, which can initiate and enhance T effector cell responses [56]. Catrine M. Persson found that CD62L plays an important role in the migration of NK cells to various inflammatory stimulations [57]. Sebelin K observed that the expression of CD86 and HLA-DR on bone marrow dendritic cells in immunosuppressed renal transplant patients decreased [58]. Chemokines and their receptors play an important role in the pathogenesis of DN, and chemokine receptor CXCR3 is involved in the release of inflammatory factors and cell damage in podocytes under high glucose [59].

In summary, we hypothesize that CD25 on IgD- CD38dim B cell, CD25 on naive-mature B cell, CD127 on granulocyte, SSC-A on HLA DR + Natural Killer, HLA DR on plasmacytoid Dendritic Cell, and HLA DR on Dendritic Cell may contribute to the onset and progression of DN through abnormal infiltration in renal tissue, leading to the production of autoantibodies, circulating immune complexes, or related inflammatory factors. These findings deepen our understanding of the interplay between immune cells and DN, offering potential insights for DN prevention strategies.

Future research requires more population data to verify the potential roles of these immune cell subsets in DN. Additionally, we encourage future studies to employ multimodal research approaches, including multi-omics analyses, basic experiments, and clinical trials, to explore how these immune cell subsets influence DN through specific molecular pathways and how these findings can be applied in clinical practice. For instance, we could develop new therapeutic strategies to enhance the function or number of these protective immune cells, or reduce the function or number of unprotective immune cells to prevent or treat DN. This might involve using specific cytokines or drugs to stimulate the production of protective immune cells, or developing vaccines to activate specific antigen-presenting pathways to increase the kidney’s resistance to diabetes-induced damage.

The study possesses several strengths. Firstly, it benefits from a large sample size, minimizing the impact of confounding factors on the results. Secondly, it provides a robust estimation of the causal relationship between exposure factors and disease, avoiding the reverse causation inherent in traditional observational studies. Thirdly, it marks the first instance of uncovering the genetic-level association between immune cells and DN. However, there are some limitations to consider. Firstly, the outcome data utilized in this study originate from the European population, limiting the generalizability of the findings. Future research should include a more diverse GWAS population to validate the results. Secondly, the available data lack detailed demographic information such as age and gender, preventing further subgroup analysis. Thirdly, to conduct sensitivity and horizontal pleiotropy analyses, more SNPs needed to be included as IVs, so instead of the traditional significance threshold (*P* < 5e-08), we chose 1e-05.

## 5. Conclusion

This study offers an initial glimpse into the genetic perspective regarding the causal relationship between immune cells and DN. It lays a critical theoretical groundwork for future pursuits in precision medicine and individualized treatment. The Myeloid Dendritic Cell emerges as a potential predictor for DN.

## Supplementary Material

Supplementary Figure 1.pdf

Supplementary Figure 2.pdf

Figures.pptx

Supplementary Table 2.xlsx

Supplementary Table 1.xlsx

Supplementary Table 3.xlsx

Change of authorship request.pdf

## Data Availability

The original contributions presented in the study are included in the article/Supplementary material, further inquiries can be directed to the corresponding author.
