# Supplementary material for: Causal relationship between 731 immune cells and the risk of diabetic nephropathy: a two‑sample bidirectional Mendelian randomization study
Source: Ren Fail. 2024 Aug 1;46(2):2387208. doi: 10.1080/0886022X.2024.2387208 (PMC11299454; doi:10.1080/0886022X.2024.2387208)

Supplementary Figure 1

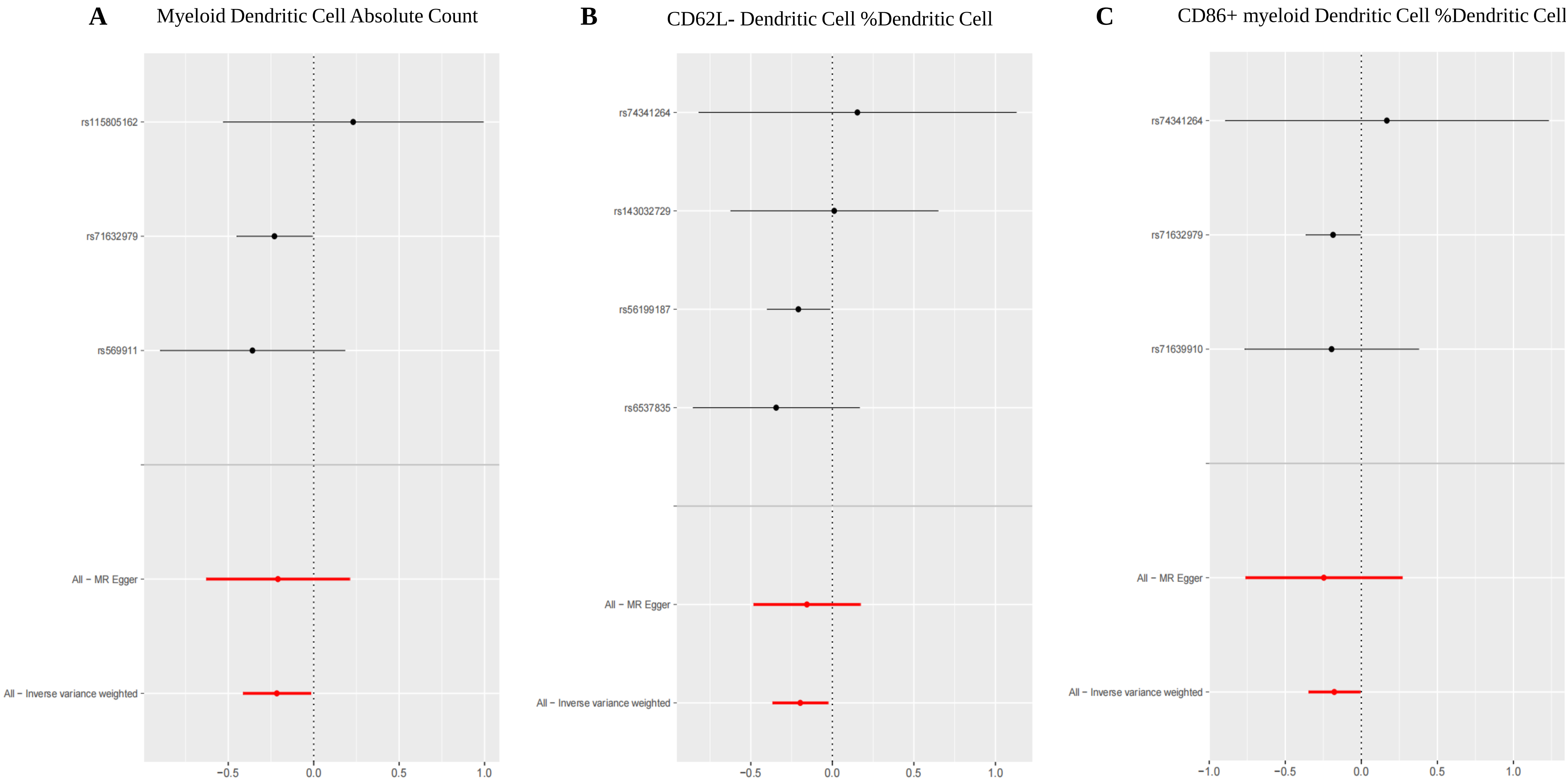

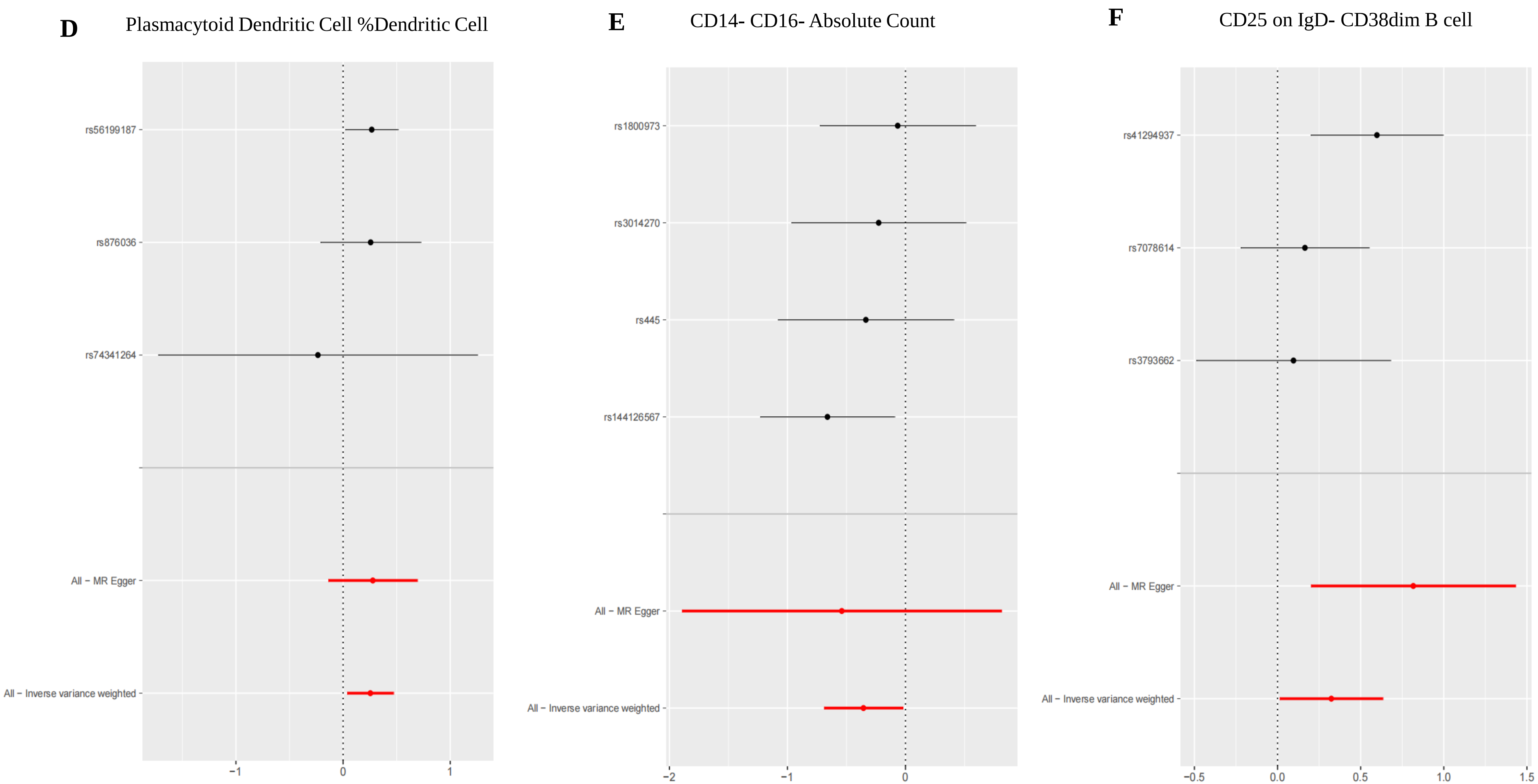

D
Plasmacytoid Dendritic Cell %Dendritic Cell
rs56199187
rs876036
rs74341264
All − MR Egger
All − Inverse variance weighted
−1
0
1
E
CD14- CD16- Absolute Count
rs1800973
rs3014270
rs445
rs144126567
All − MR Egger
All − Inverse variance weighted
−2
−1
0
F
CD25 on IgD- CD38dim B cell
rs41294937
rs7078614
rs3793662
All − MR Egger
All − Inverse variance weighted
−0.5
0.0
0.5
1.0
1.5

G CD25 on naive-mature B cell

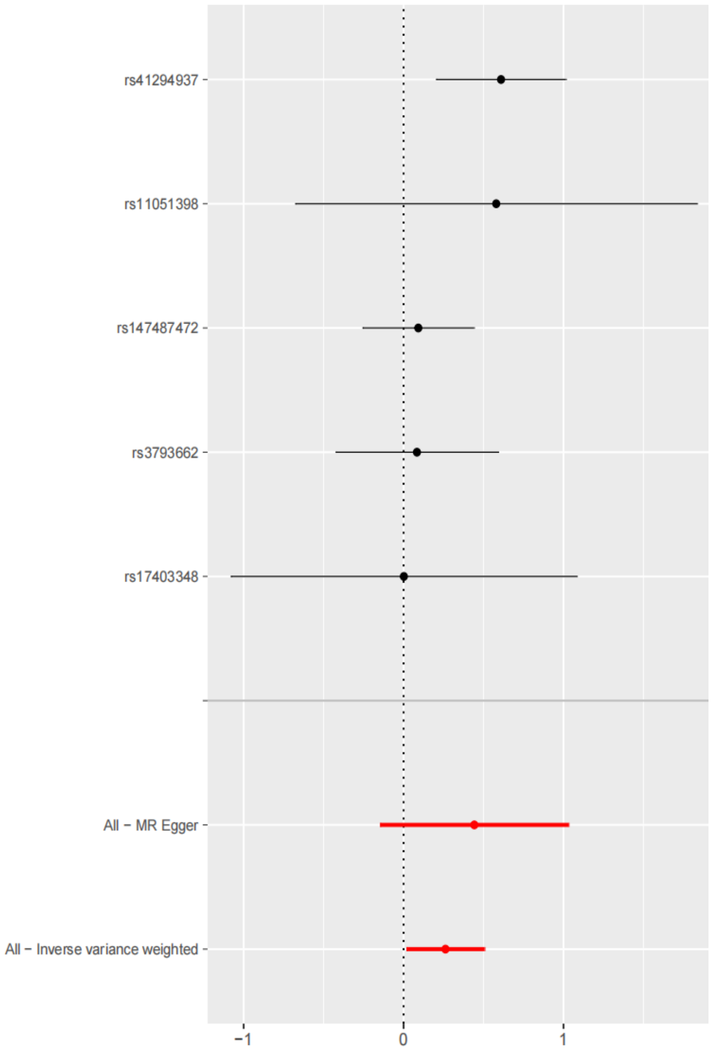

H CD3 on CD28+ CD45RA- CD8+ T cell

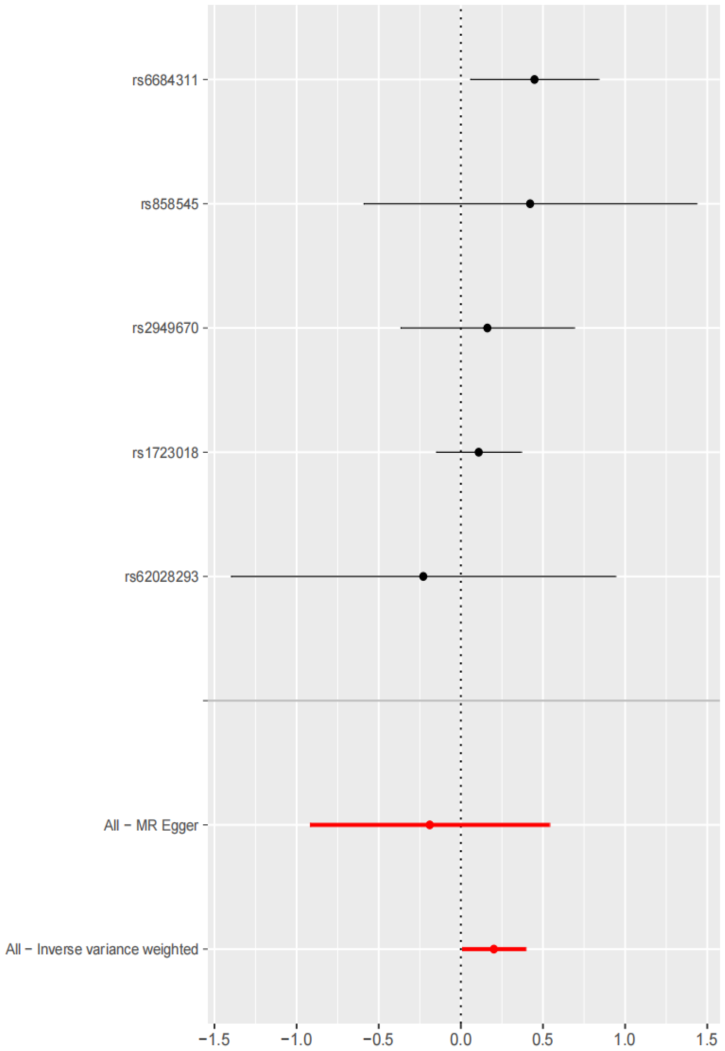

I CD127 on granulocyte

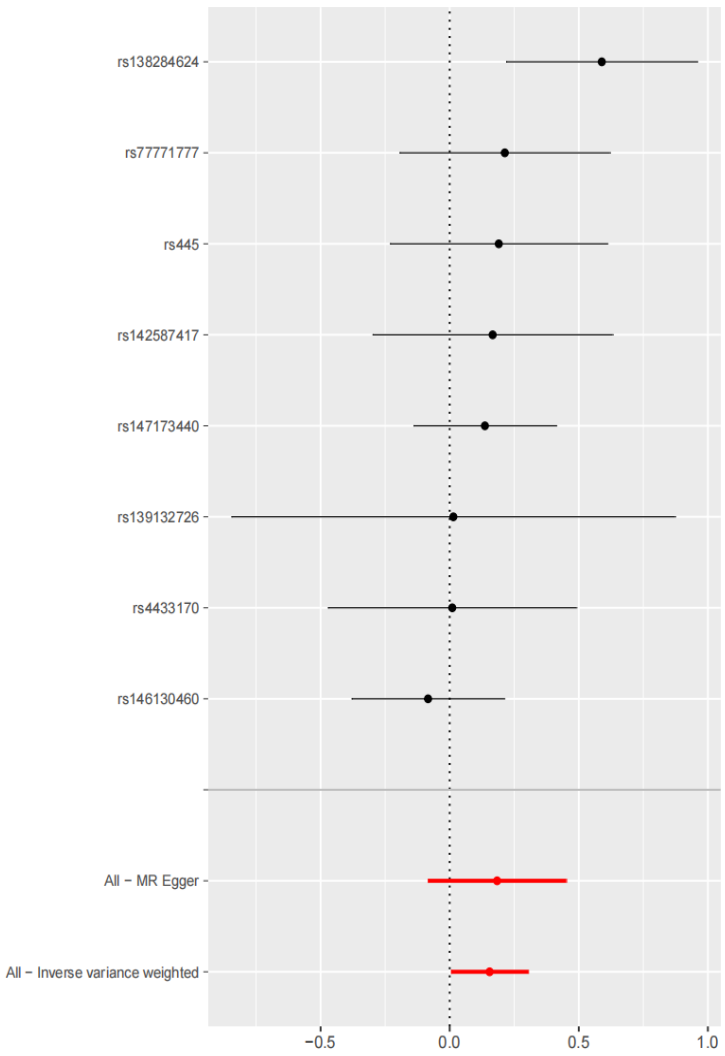

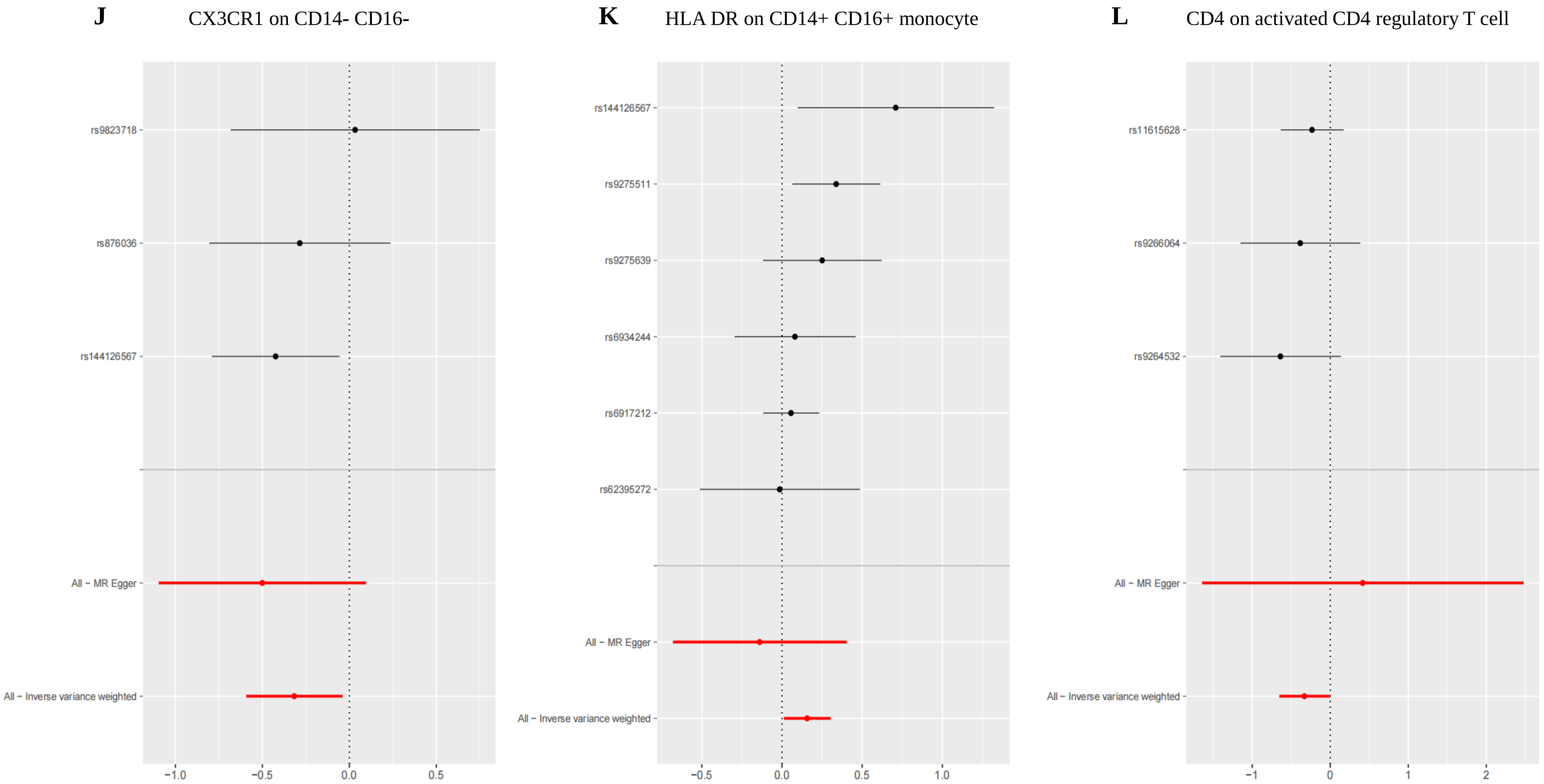
J
CX3CR1 on CD14- CD16-
rs9823718
rs876036
rs144126567
All − MR Egger
All − Inverse variance weighted
−1.0
−0.5
0.0
0.5
K
HLA DR on CD14+ CD16+ monocyte
rs144126567
rs9275511
rs9275639
rs6934244
rs6917212
rs62395272
All − MR Egger
All − Inverse variance weighted
−0.5
0.0
0.5
1.0
L
CD4 on activated CD4 regulatory T cell
rs11615628
rs9266064
rs9264532
All − MR Egger
All − Inverse variance weighted
−1
0
1
2

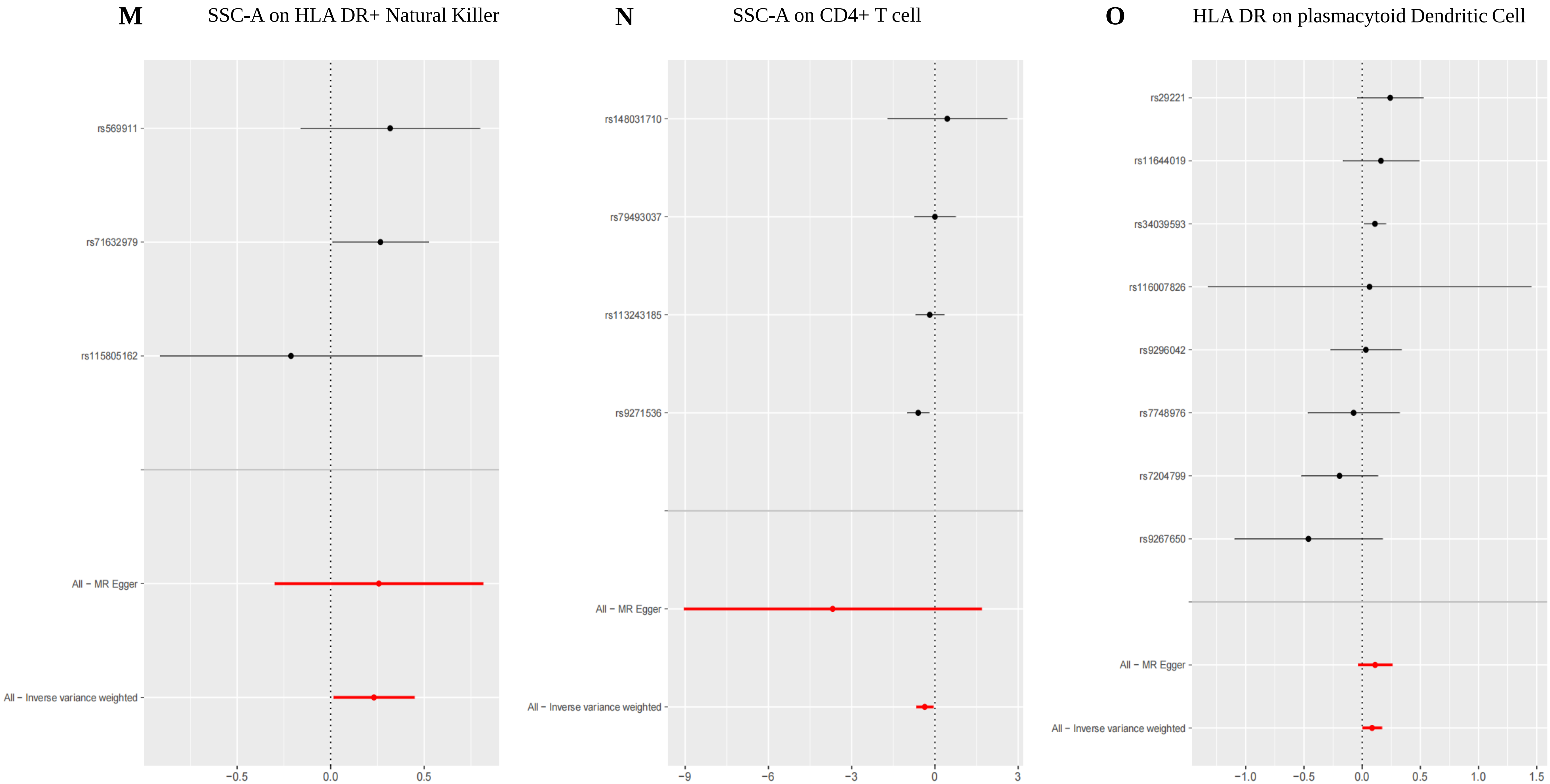
M
SSC-A on HLA DR+ Natural Killer
rs569911
rs71632979
rs115805162
All − MR Egger
All − Inverse variance weighted
−0.5
0.0
0.5
N
SSC-A on CD4+ T cell
rs148031710
rs79493037
rs113243185
rs9271536
All − MR Egger
All − Inverse variance weighted
−9
−6
−3
0
3
O
HLA DR on plasmacytoid Dendritic Cell
rs29221
rs11644019
rs34039593
rs116007826
rs9296042
rs7748976
rs7204799
rs9267650
All − MR Egger
All − Inverse variance weighted
−1.0
−0.5
0.0
0.5
1.0
1.5

P HLA DR on Dendritic Cell

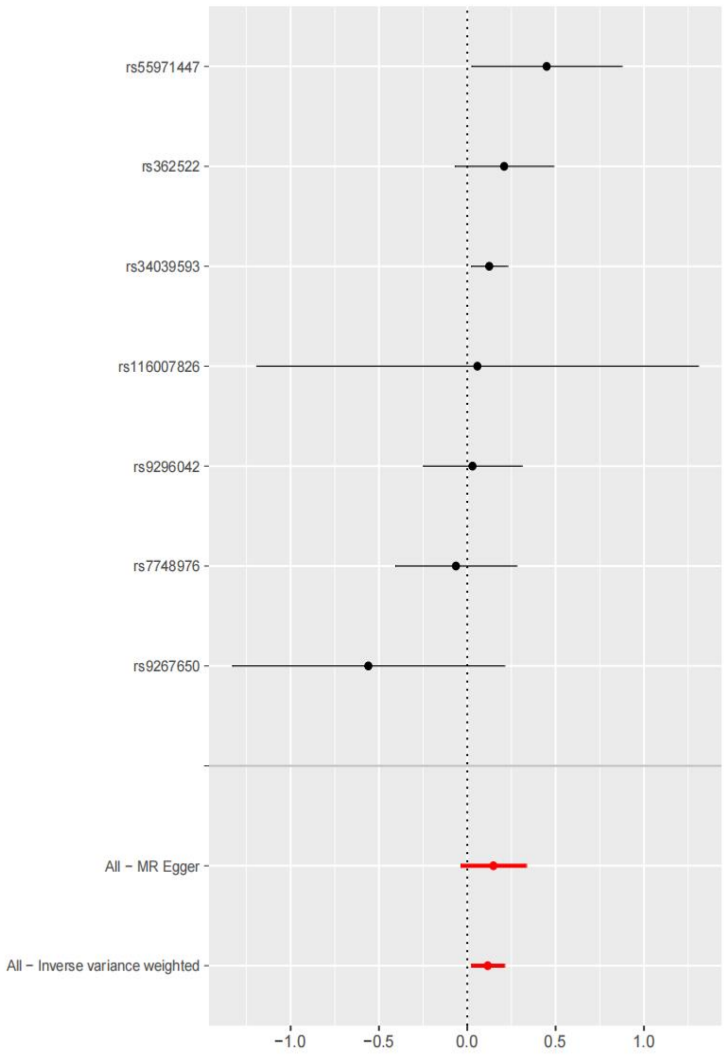

Supplement: Supplementary Figure 1.pdf [file IRNF_A_2387208_SM4109.pdf]
