# Supplementary material for: Causal relationship between 731 immune cells and the risk of diabetic nephropathy: a two‑sample bidirectional Mendelian randomization study
Source: Ren Fail. 2024 Aug 1;46(2):2387208. doi: 10.1080/0886022X.2024.2387208 (PMC11299454; doi:10.1080/0886022X.2024.2387208)
Supplement: Supplementary Figure 2.pdf [file IRNF_A_2387208_SM4108.pdf]

Supplementary Figure 2

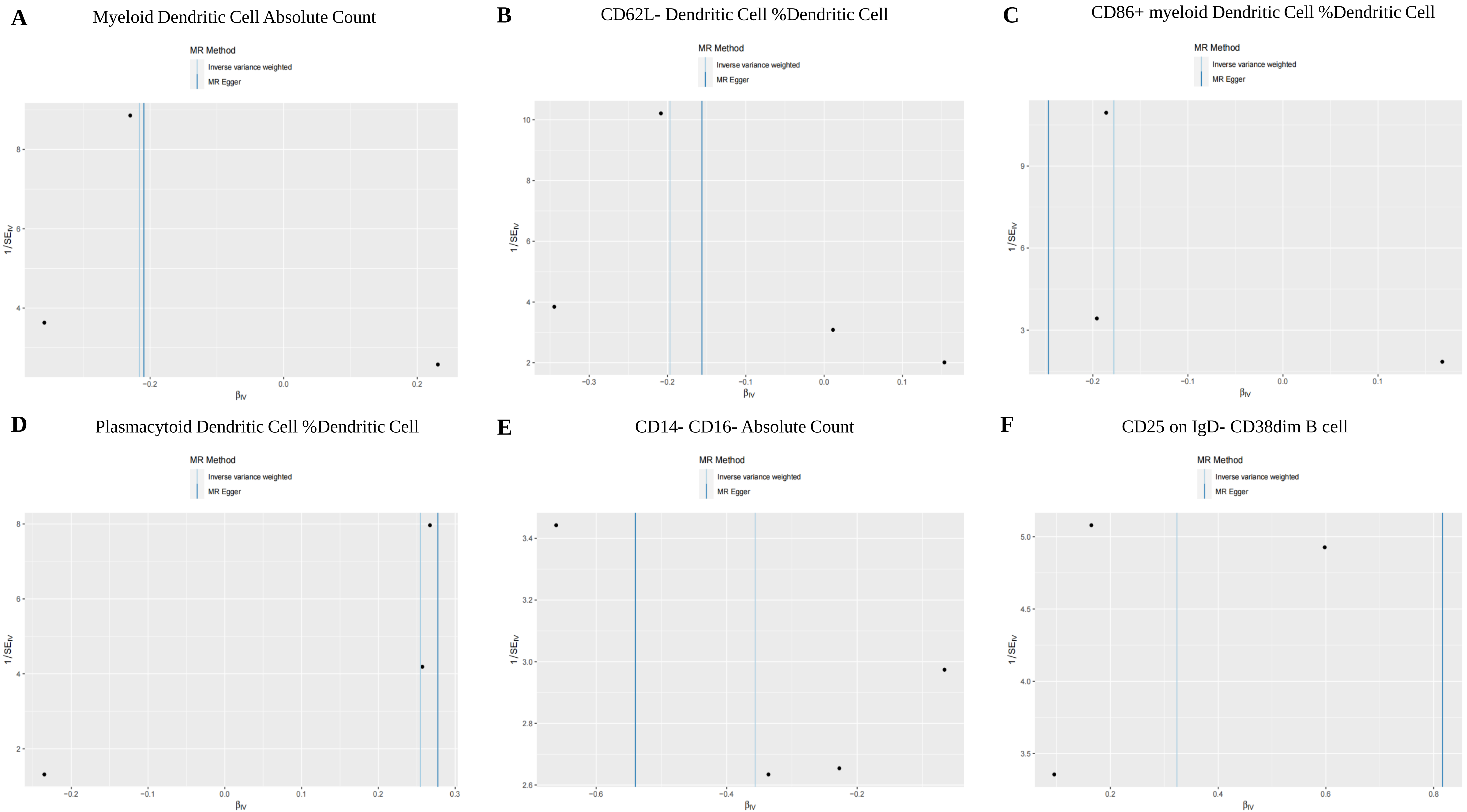

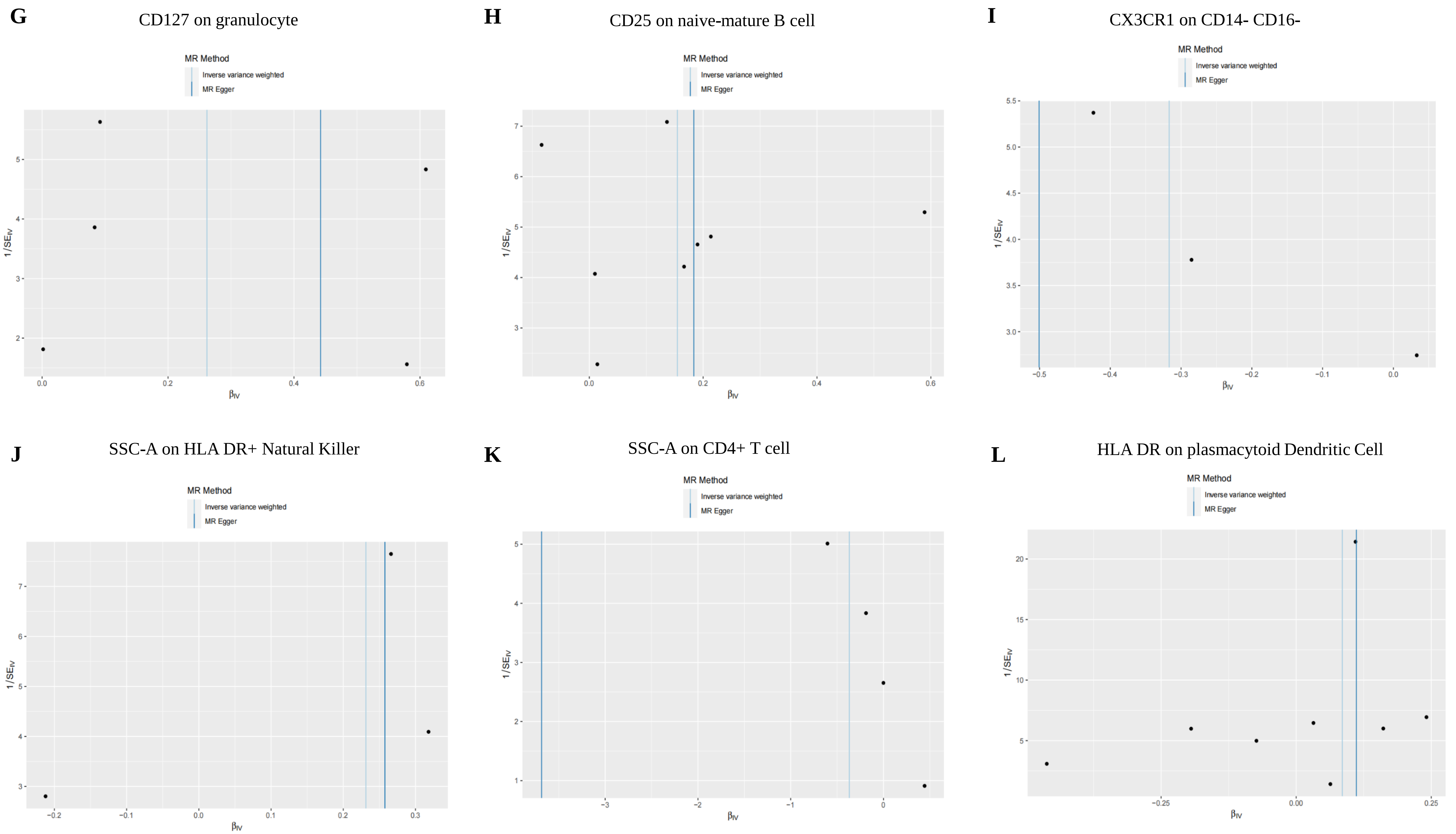
G
CD127 on granulocyte
H
CD25 on naive-mature B cell
I
CX3CR1 on CD14- CD16-
J
SSC-A on HLA DR+ Natural Killer
K
SSC-A on CD4+ T cell
L
HLA DR on plasmacytoid Dendritic Cell
MR Method
Inverse variance weighted
MR Egger
1/SE$_{IV}$
β_{IV}
