# Supplementary material for: Causal relationship between 731 immune cells and the risk of diabetic nephropathy: a two‑sample bidirectional Mendelian randomization study
Source: Ren Fail. 2024 Aug 1;46(2):2387208. doi: 10.1080/0886022X.2024.2387208 (PMC11299454; doi:10.1080/0886022X.2024.2387208)
Supplement: Figures.pptx [file IRNF_A_2387208_SM4107.pptx]

## Slide 1
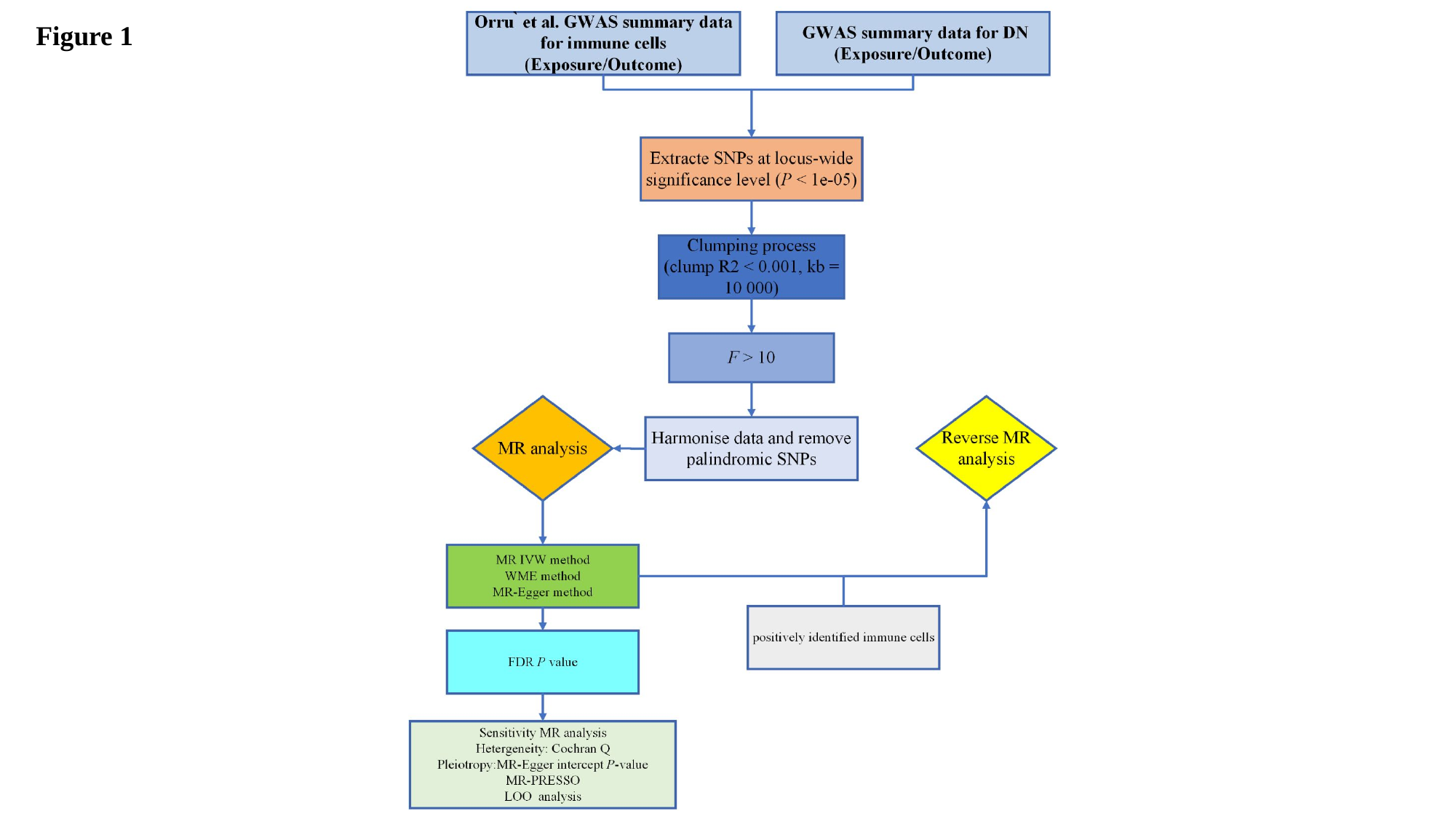

Figure 1
Figure 1

## Slide 2
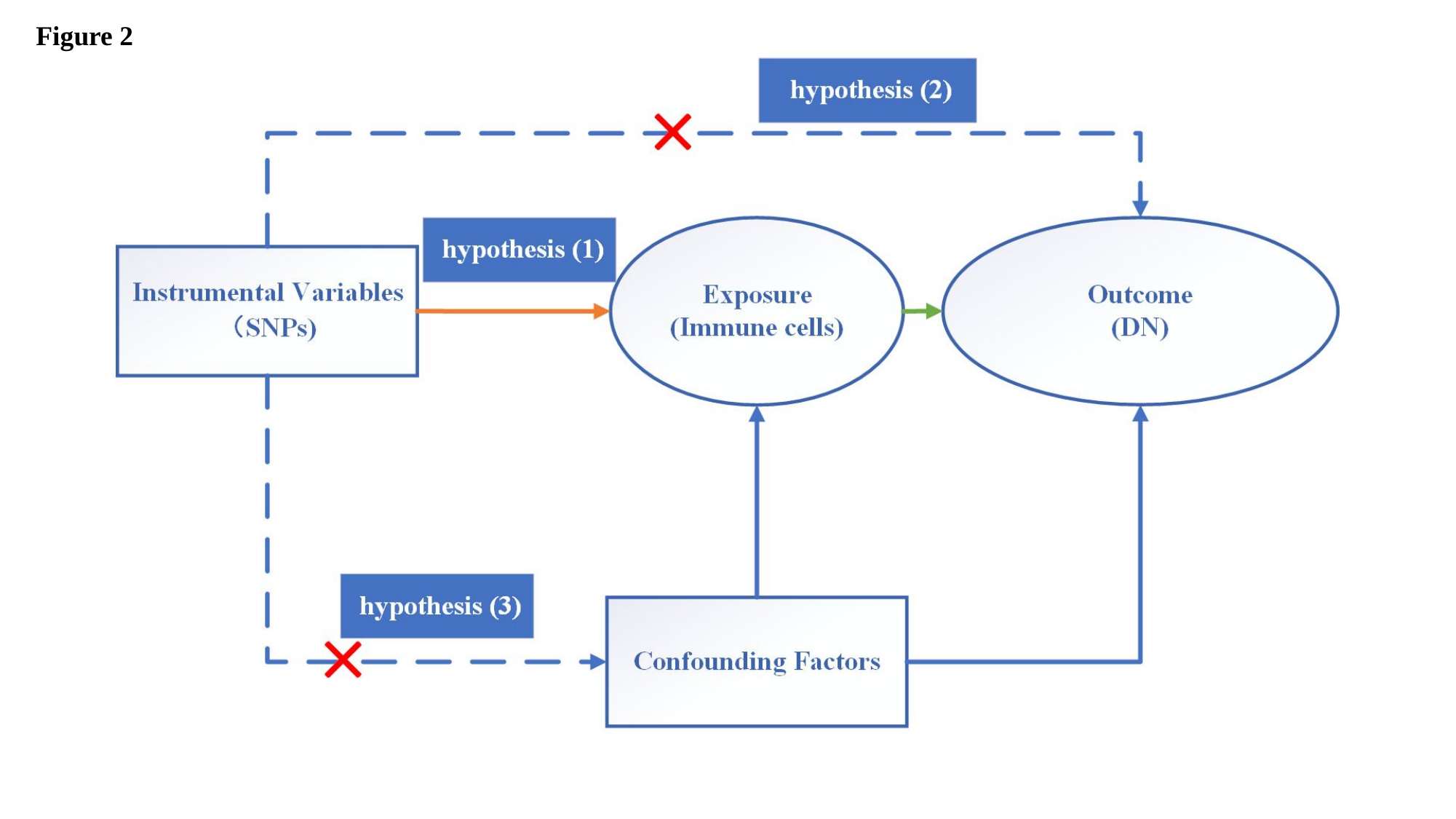

Figure 2
Figure 2

## Slide 3
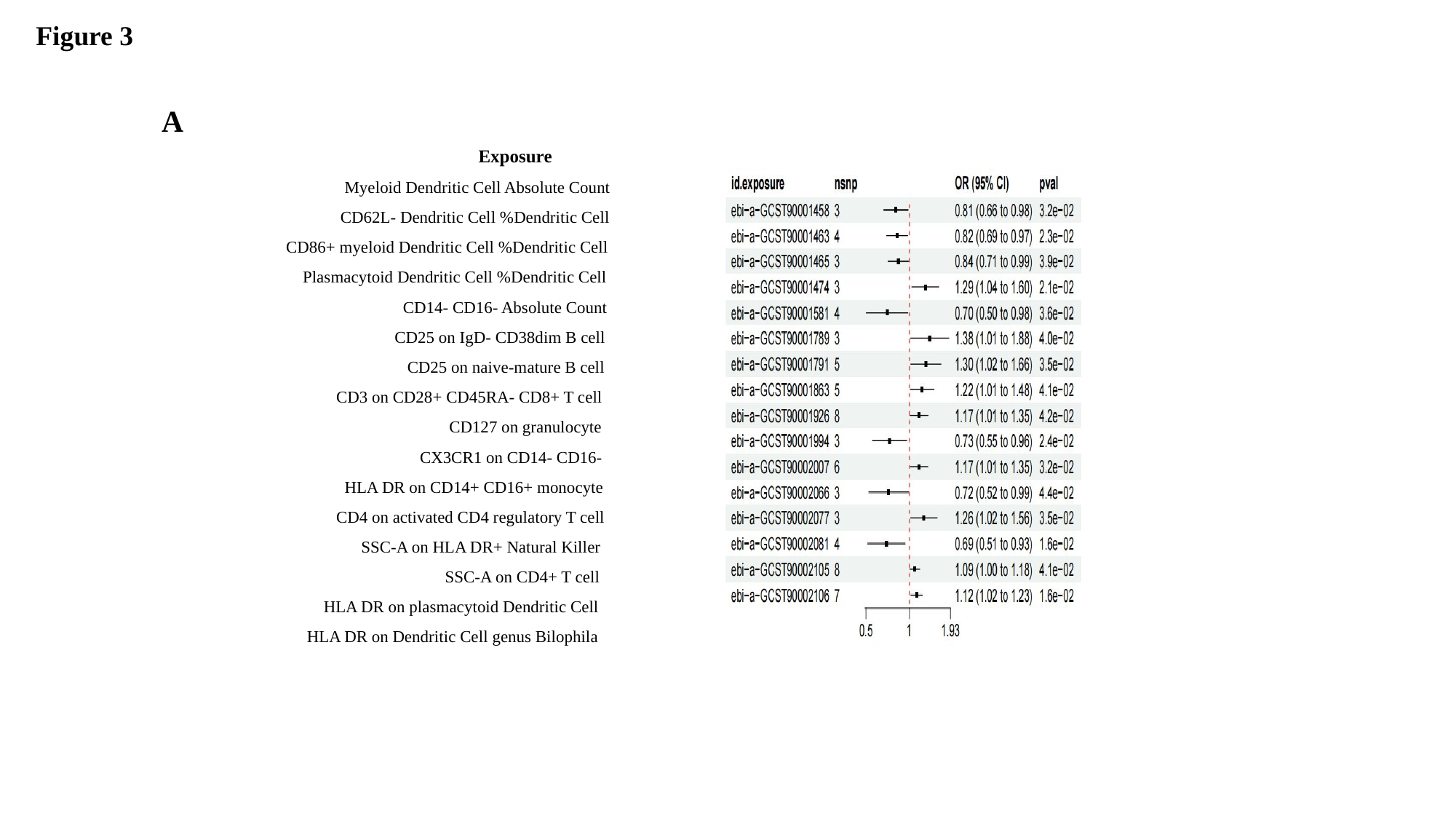

Figure 3
 Exposure
 Myeloid Dendritic Cell Absolute Count
 CD62L- Dendritic Cell %Dendritic Cell
 CD86+ myeloid Dendritic Cell %Dendritic Cell
 Plasmacytoid Dendritic Cell %Dendritic Cell
 CD14- CD16- Absolute Count
 CD25 on IgD- CD38dim B cell
 CD25 on naive-mature B cell
 CD3 on CD28+ CD45RA- CD8+ T cell
 CD127 on granulocyte
 CX3CR1 on CD14- CD16-
 HLA DR on CD14+ CD16+ monocyte
 CD4 on activated CD4 regulatory T cell
 SSC-A on HLA DR+ Natural Killer
 SSC-A on CD4+ T cell
 HLA DR on plasmacytoid Dendritic Cell
 HLA DR on Dendritic Cell genus Bilophila
A

## Slide 4
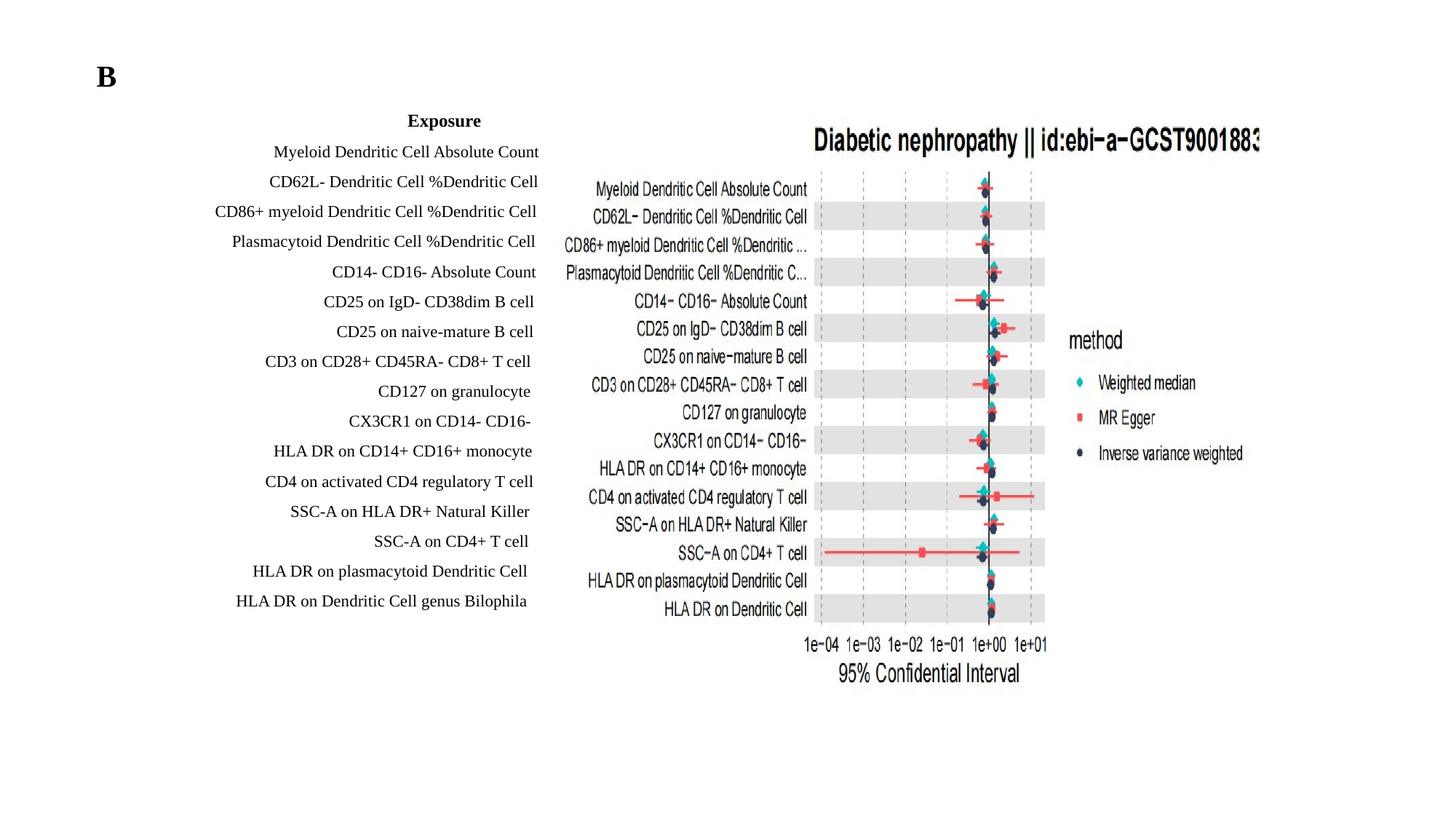

Figure 3
B
 Exposure
 Myeloid Dendritic Cell Absolute Count
 CD62L- Dendritic Cell %Dendritic Cell
 CD86+ myeloid Dendritic Cell %Dendritic Cell
 Plasmacytoid Dendritic Cell %Dendritic Cell
 CD14- CD16- Absolute Count
 CD25 on IgD- CD38dim B cell
 CD25 on naive-mature B cell
 CD3 on CD28+ CD45RA- CD8+ T cell
 CD127 on granulocyte
 CX3CR1 on CD14- CD16-
 HLA DR on CD14+ CD16+ monocyte
 CD4 on activated CD4 regulatory T cell
 SSC-A on HLA DR+ Natural Killer
 SSC-A on CD4+ T cell
 HLA DR on plasmacytoid Dendritic Cell
 HLA DR on Dendritic Cell genus Bilophila
A

## Slide 5
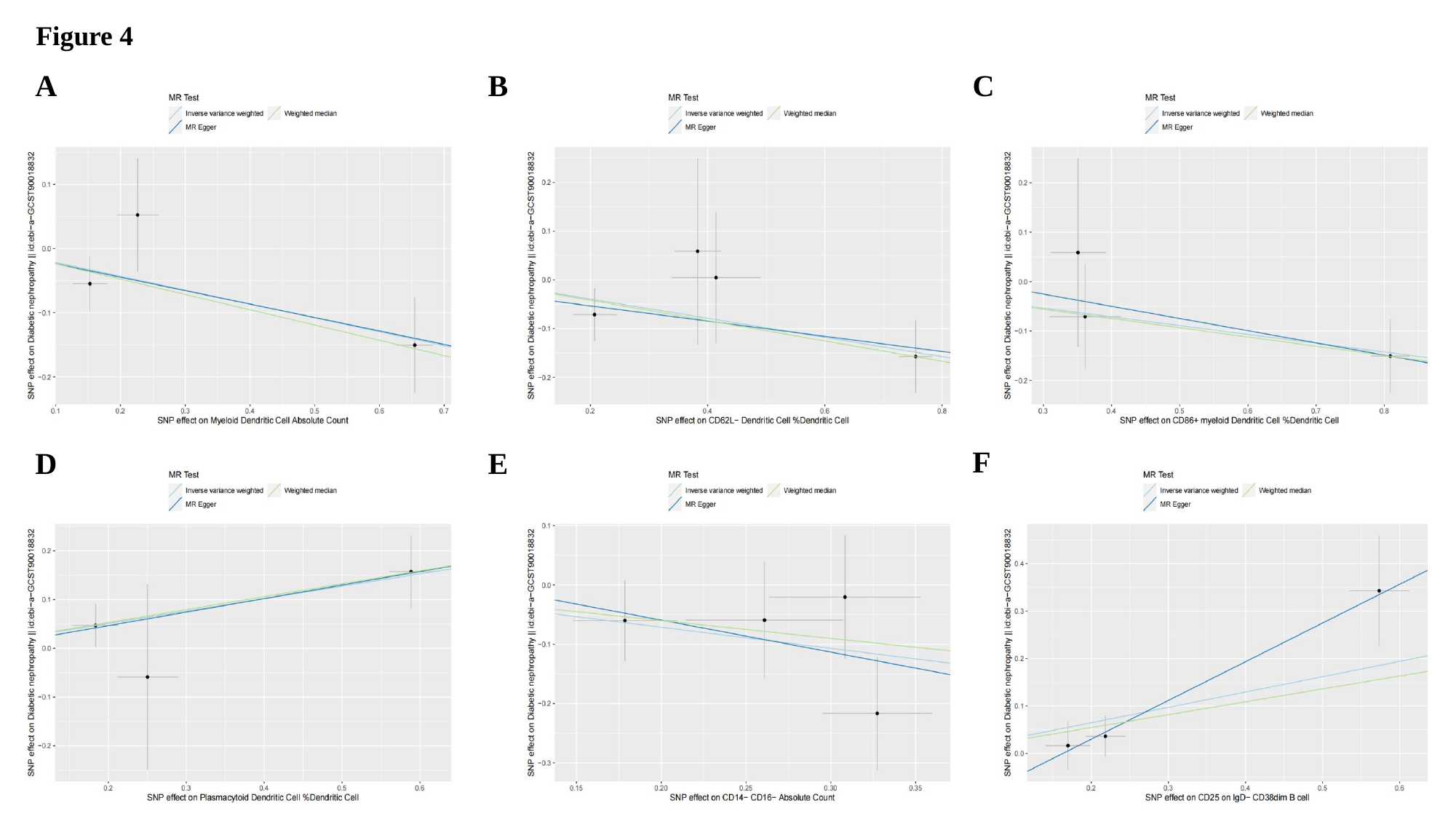

Figure 4
Figure 4
A
B
C
A
F
D
E

## Slide 6
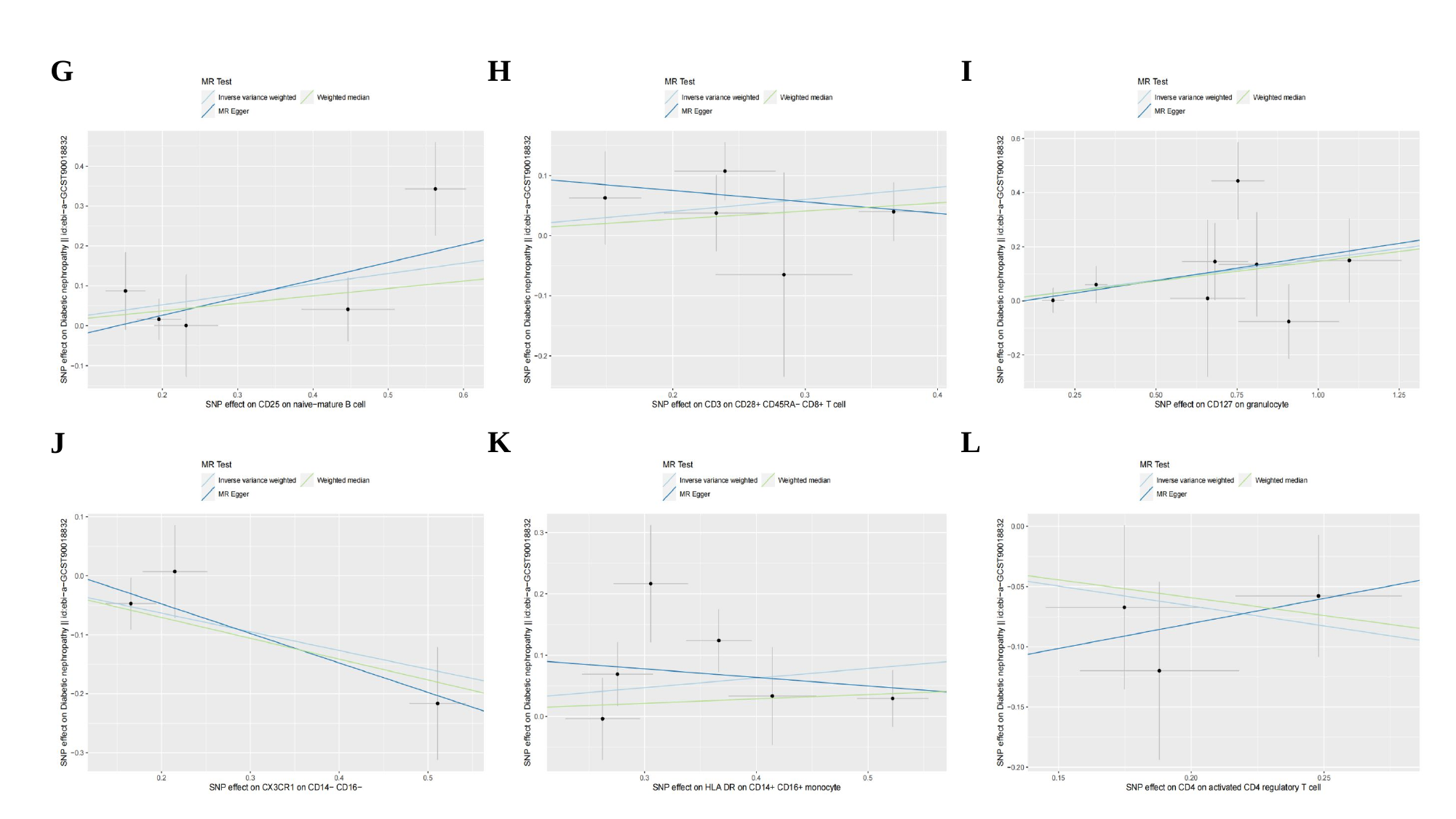

G
H
I
G
J
K
L
J

## Slide 7
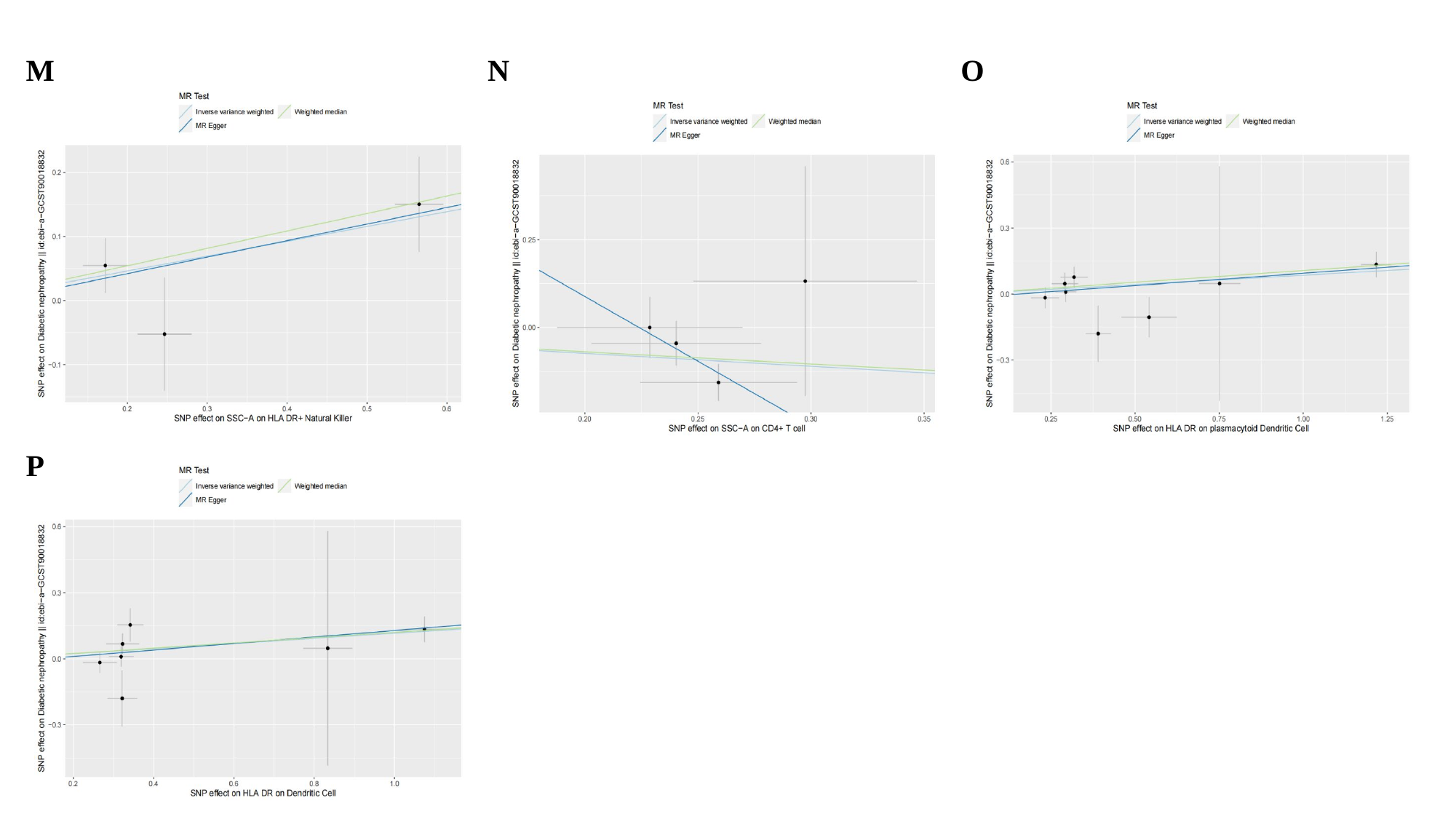

M
N
O
M
P
P

## Slide 8
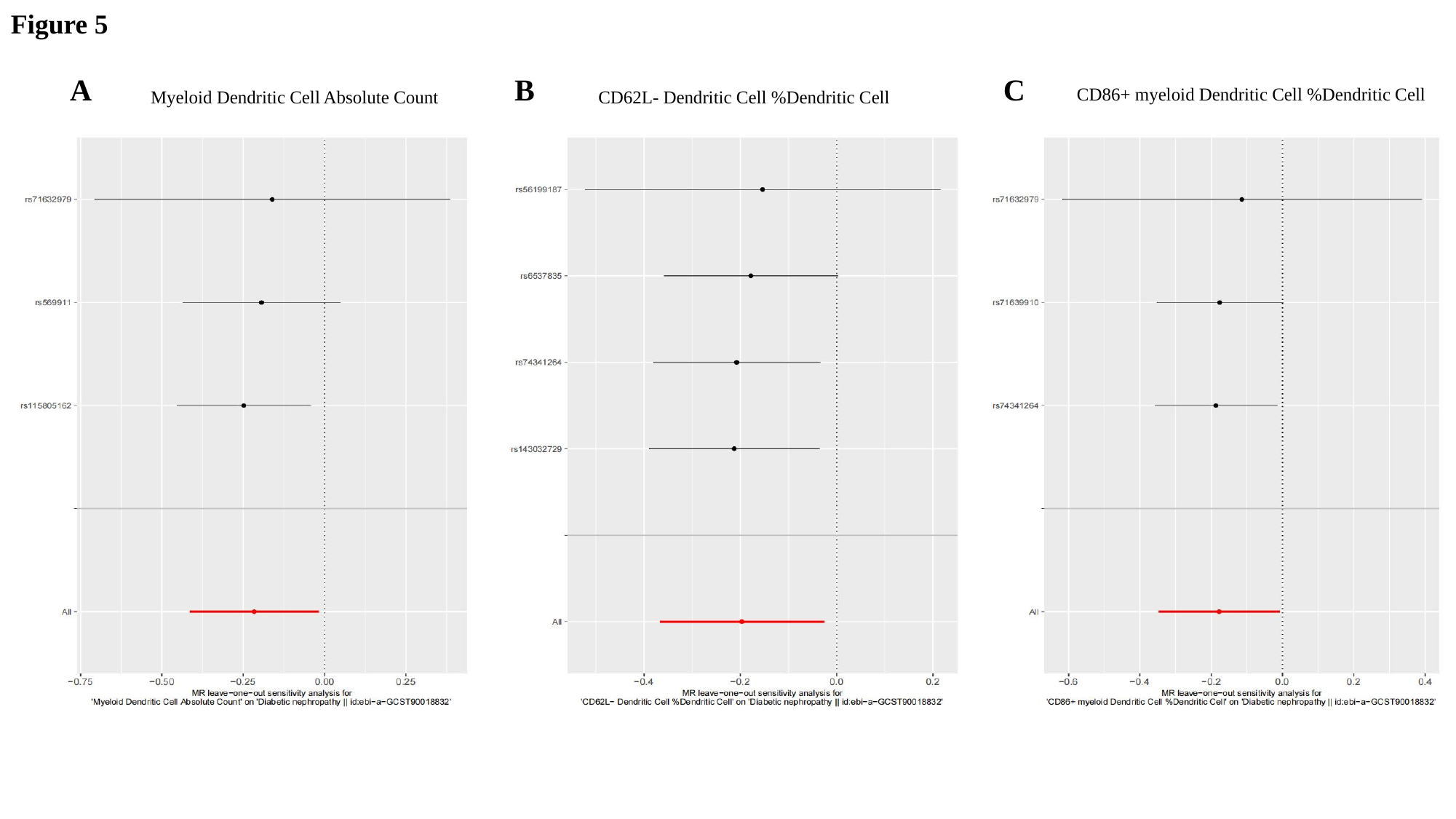

Figure 5
Figure 5
A
A
B
C
CD86+ myeloid Dendritic Cell %Dendritic Cell
CD86+ myeloid Dendritic Cell %Dendritic Cell
Myeloid Dendritic Cell Absolute Count
CD62L- Dendritic Cell %Dendritic Cell

## Slide 9
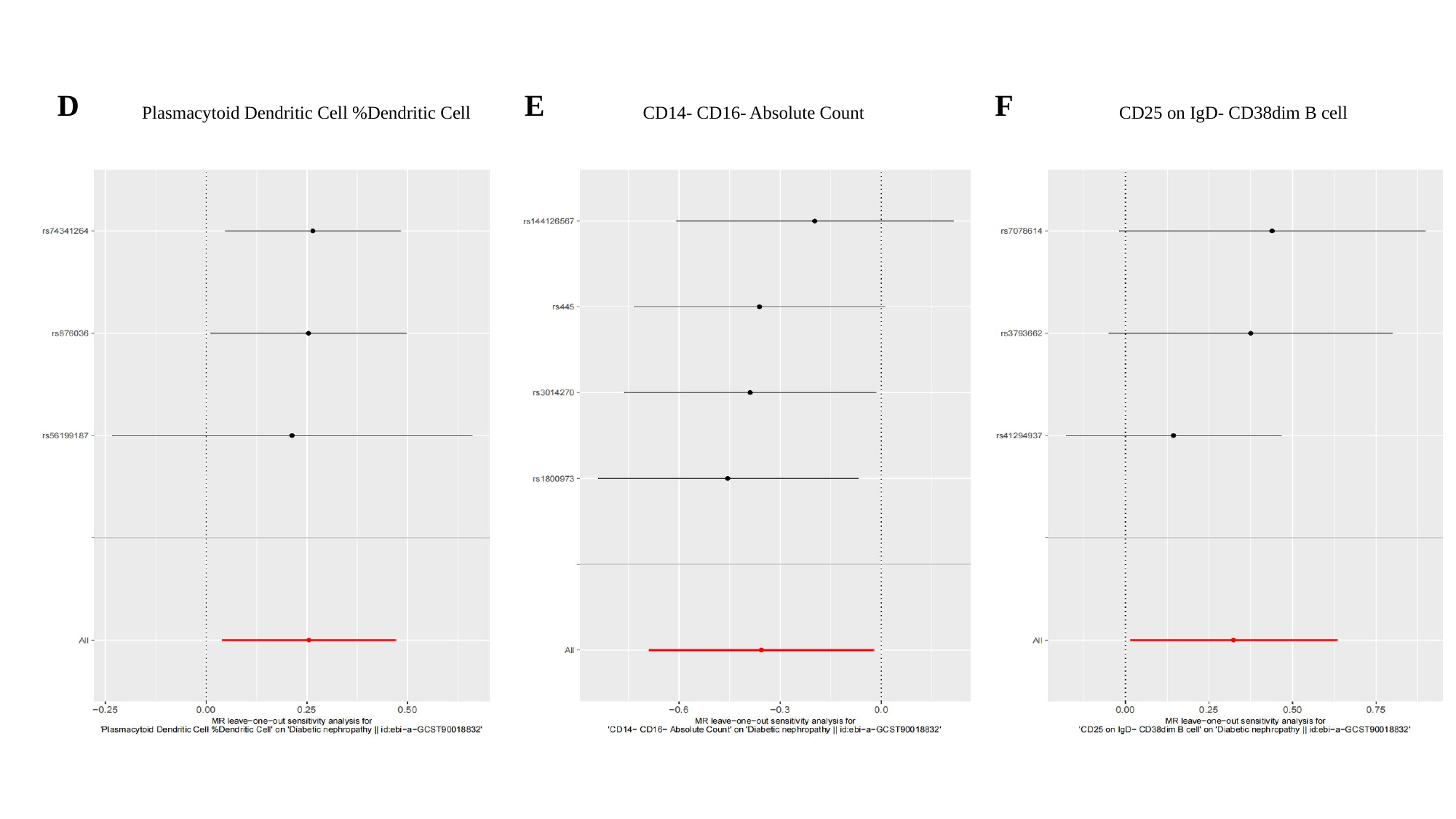

D
D
E
F
CD25 on IgD- CD38dim B cell
Plasmacytoid Dendritic Cell %Dendritic Cell
CD14- CD16- Absolute Count
CD25 on IgD- CD38dim B cell

## Slide 10
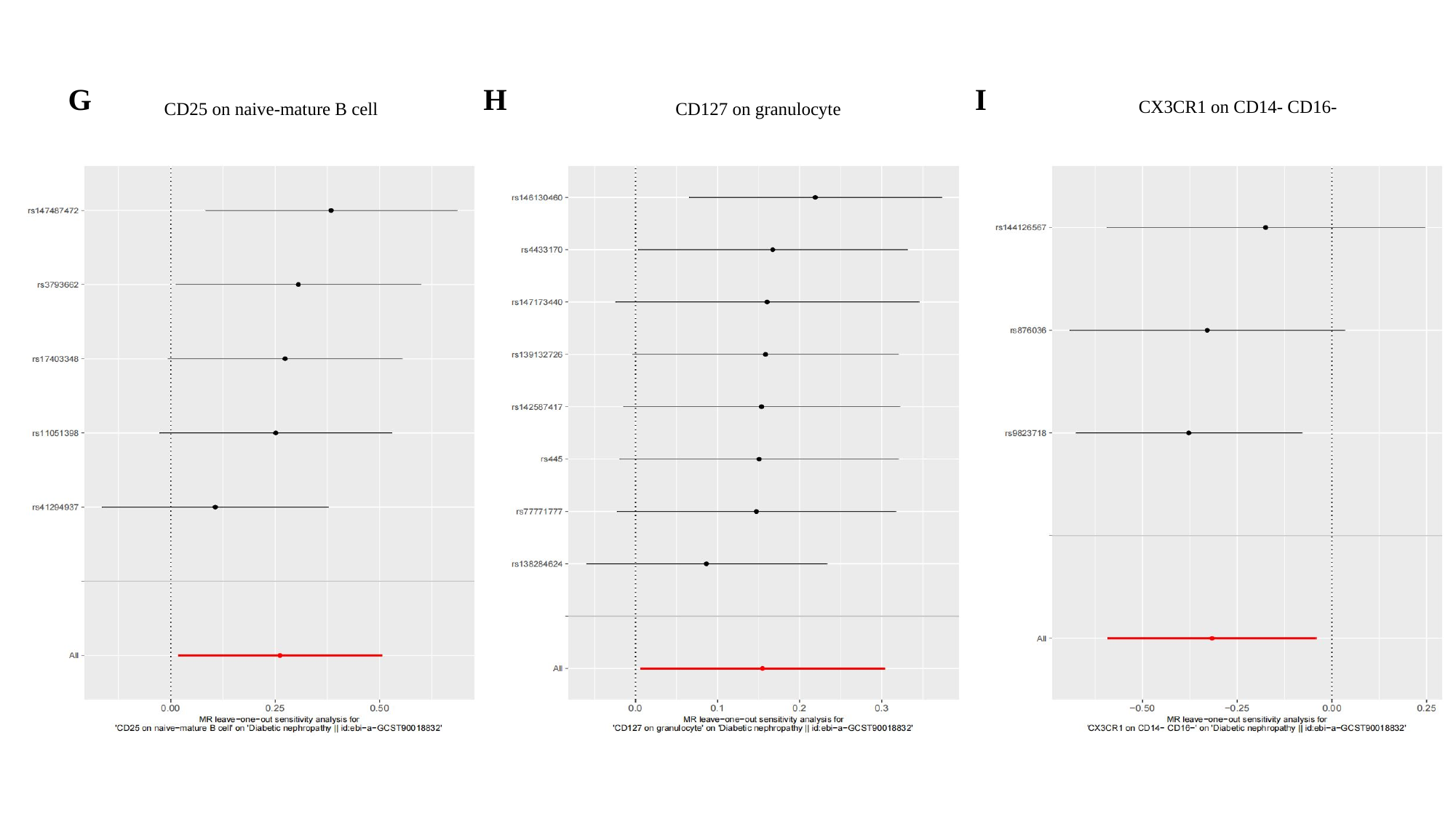

G
G
H
I
CX3CR1 on CD14- CD16-
CX3CR1 on CD14- CD16-
	CD127 on granulocyte
CD25 on naive-mature B cell

## Slide 11
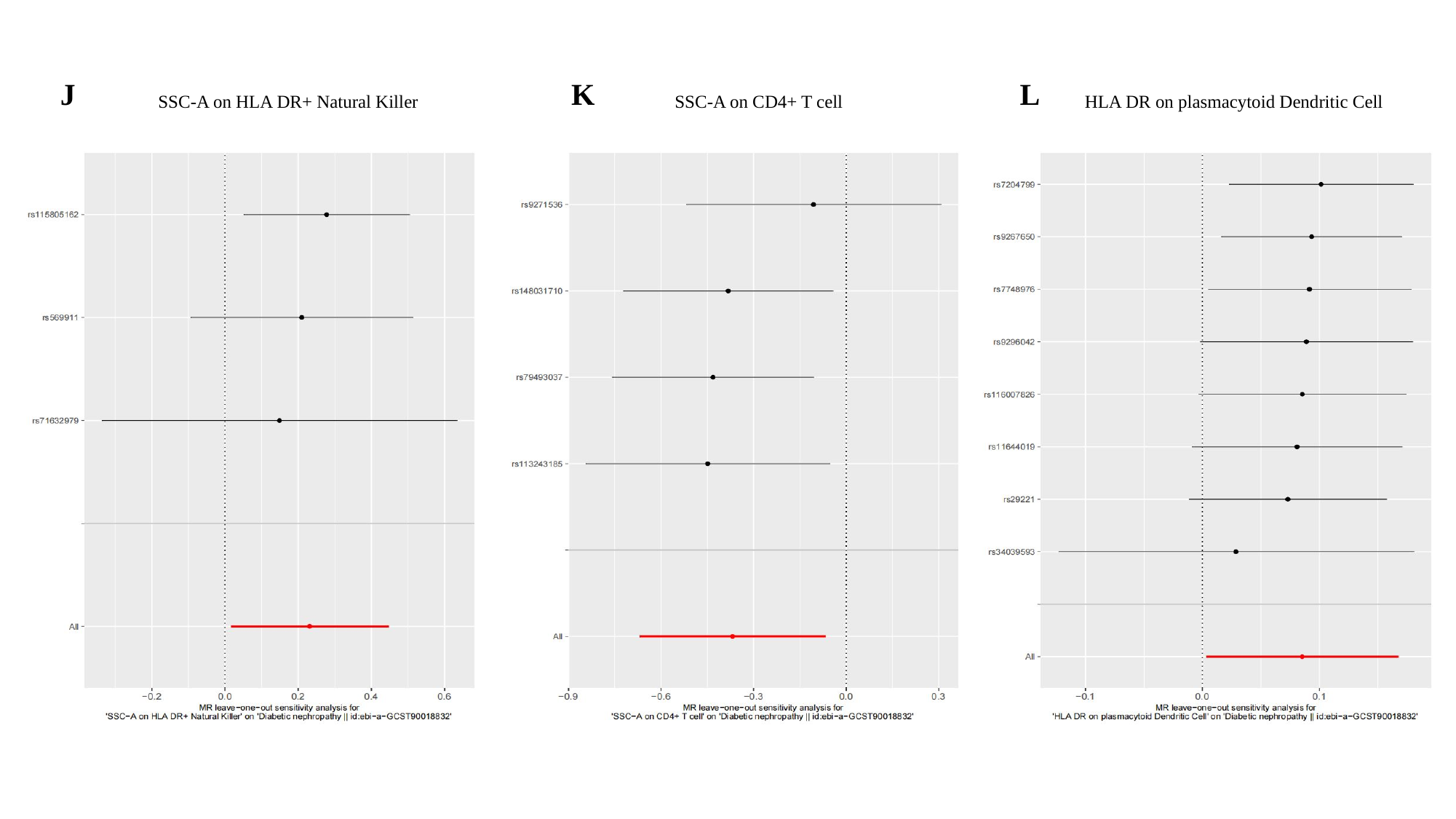

J
J
K
L
SSC-A on HLA DR+ Natural Killer
HLA DR on plasmacytoid Dendritic Cell
SSC-A on CD4+ T cell
HLA DR on plasmacytoid Dendritic Cell

## Slide 12
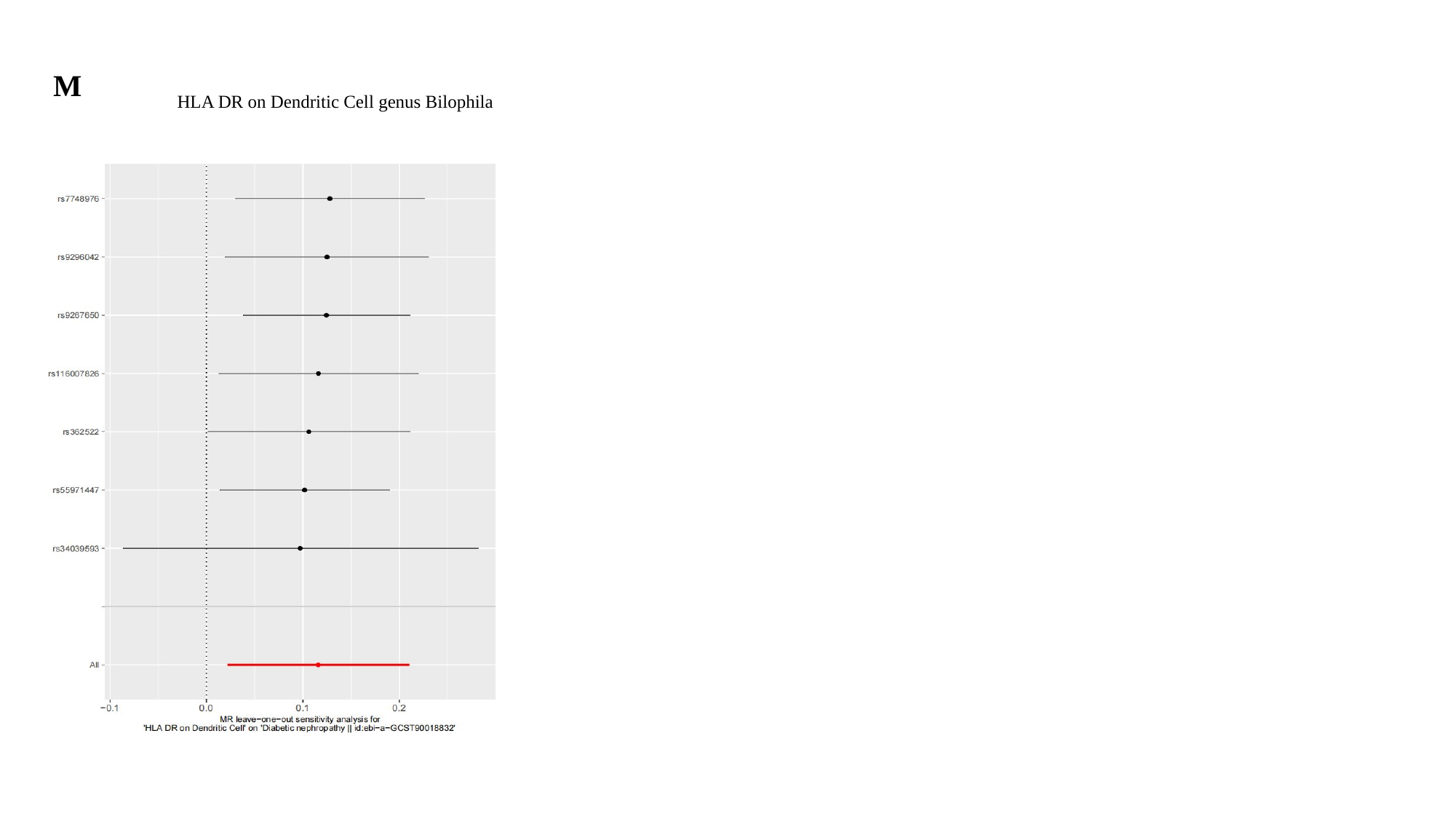

M
M
	HLA DR on Dendritic Cell genus Bilophila

## Slide 13
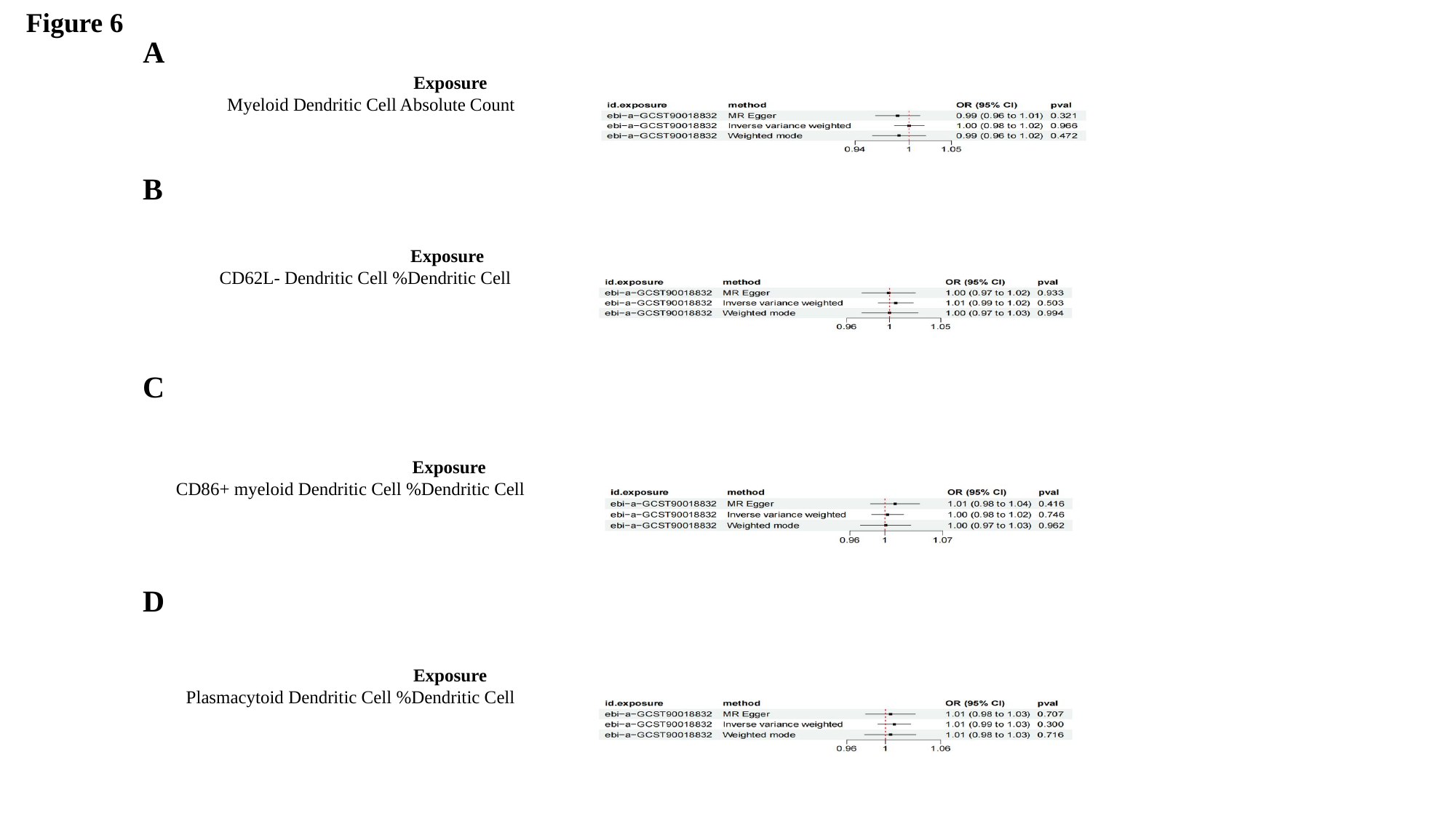

Figure 6
A
 Exposure
Myeloid Dendritic Cell Absolute Count
B
 Exposure
CD62L- Dendritic Cell %Dendritic Cell
C
 Exposure
CD86+ myeloid Dendritic Cell %Dendritic Cell
D
 Exposure
Plasmacytoid Dendritic Cell %Dendritic Cell

## Slide 14
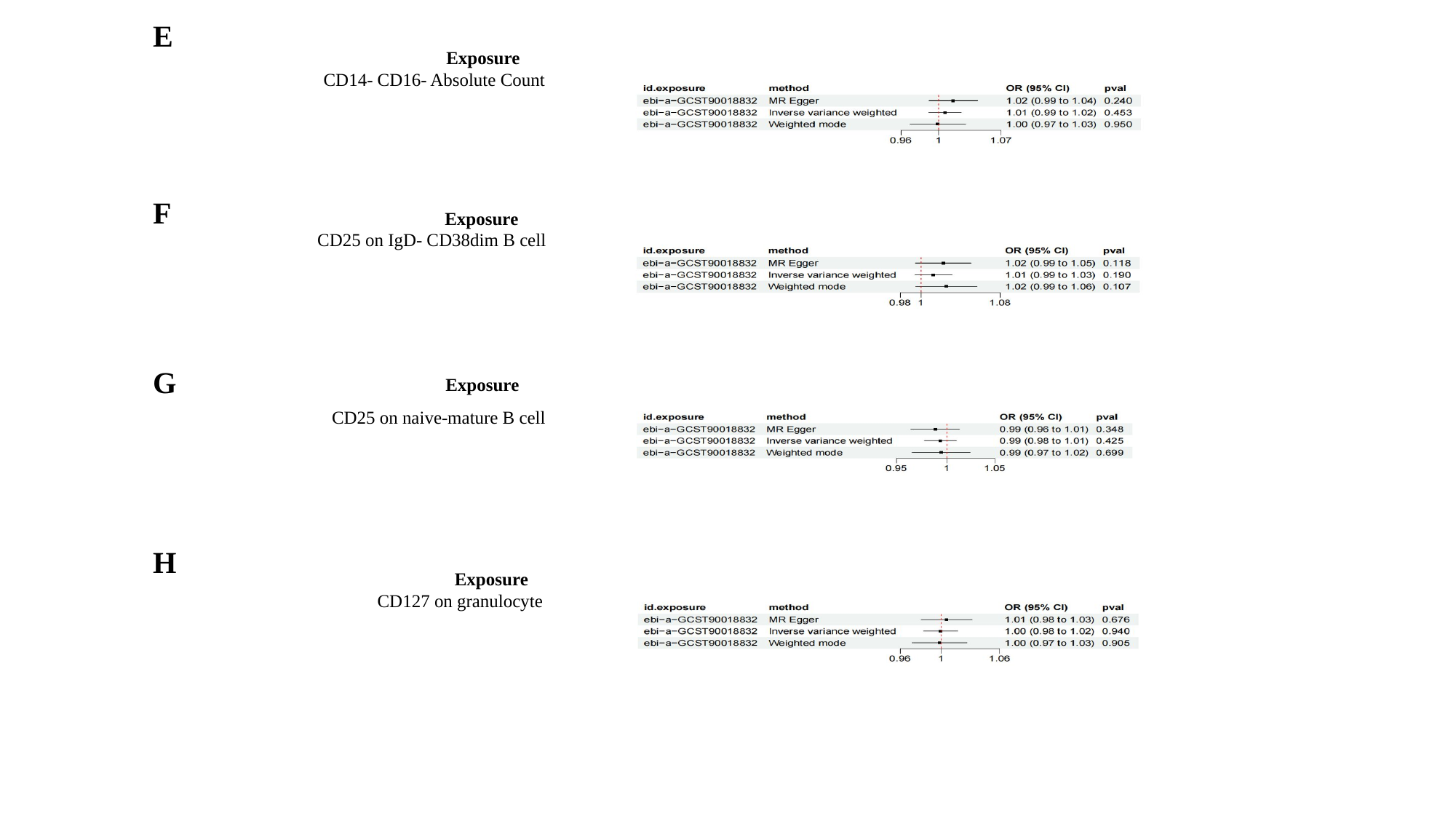

E
 Exposure
 CD14- CD16- Absolute Count
E
 Exposure
 CD25 on IgD- CD38dim B cell
F
F
 Exposure
 CD25 on naive-mature B cell
G
G
H
 Exposure
 CD127 on granulocyte
H

## Slide 15
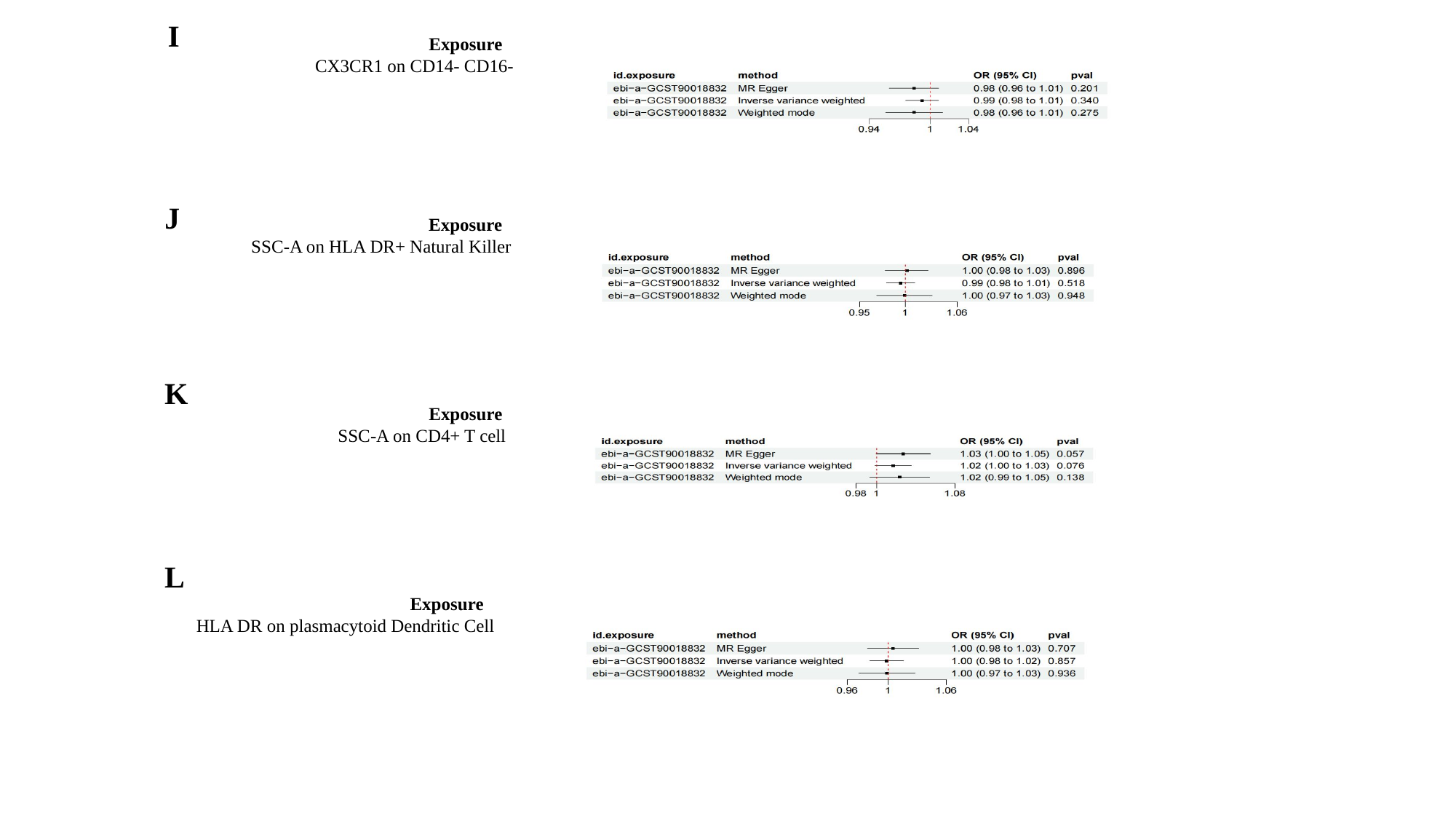

Exposure
 CX3CR1 on CD14- CD16-
I
I
 Exposure
SSC-A on HLA DR+ Natural Killer
J
J
K
 Exposure
 SSC-A on CD4+ T cell
K
L
 Exposure
HLA DR on plasmacytoid Dendritic Cell
L

## Slide 16
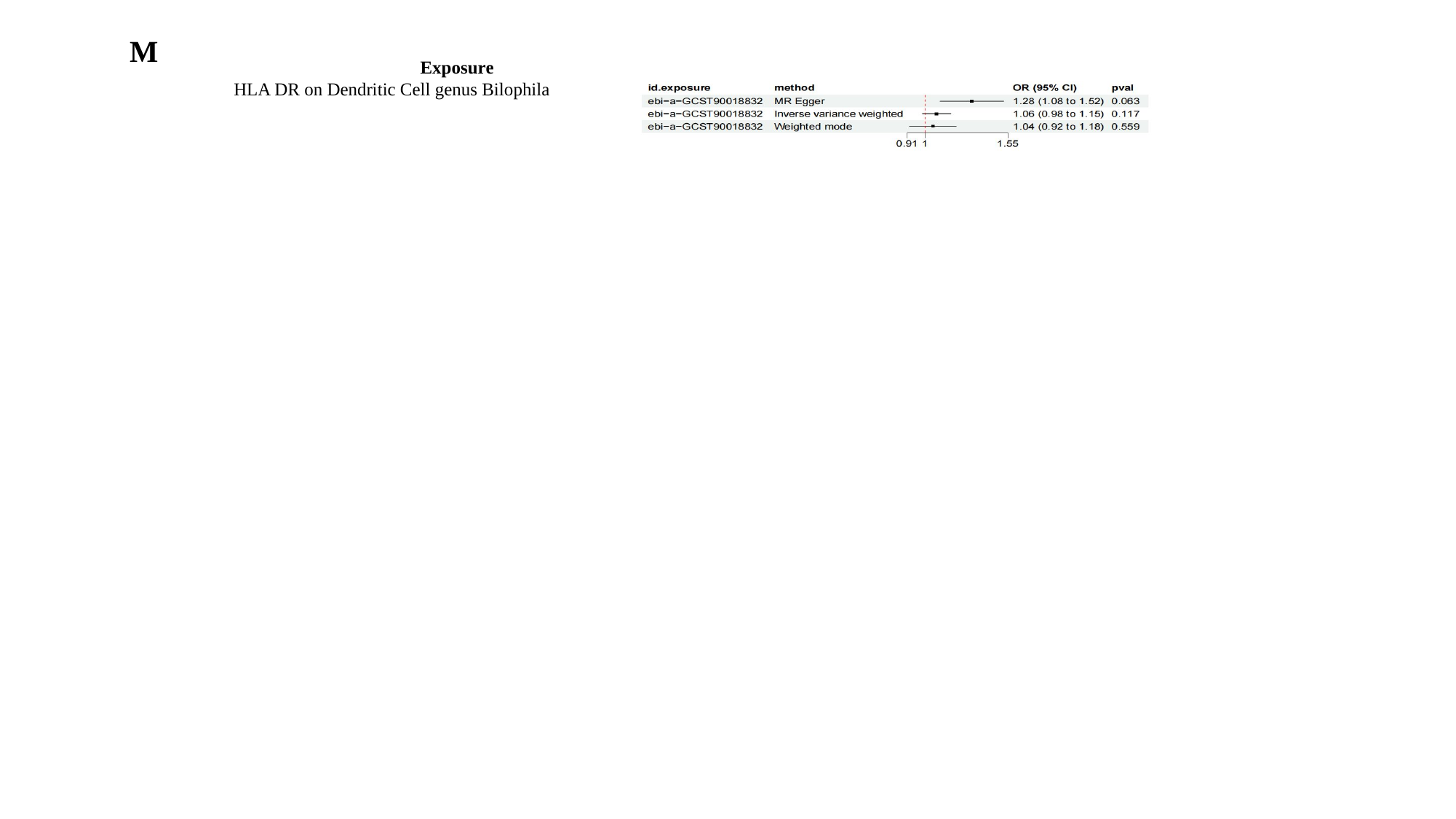

Exposure
HLA DR on Dendritic Cell genus Bilophila
M
M

## Slide 17
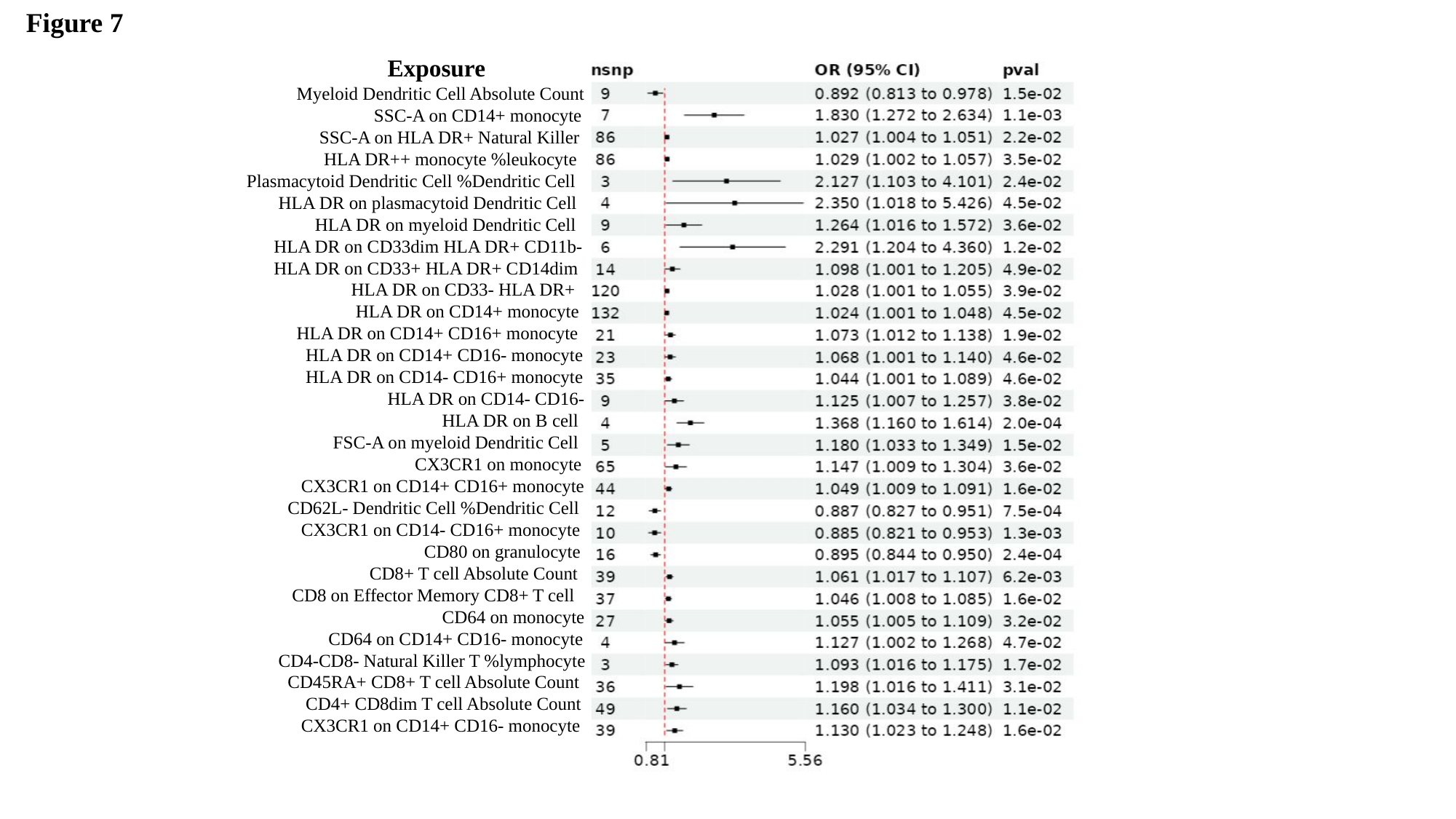

Figure 7
 Exposure
 Myeloid Dendritic Cell Absolute Count
 SSC-A on CD14+ monocyte
 SSC-A on HLA DR+ Natural Killer
 HLA DR++ monocyte %leukocyte
 Plasmacytoid Dendritic Cell %Dendritic Cell
 HLA DR on plasmacytoid Dendritic Cell
 HLA DR on myeloid Dendritic Cell
 HLA DR on CD33dim HLA DR+ CD11b-
 HLA DR on CD33+ HLA DR+ CD14dim
 HLA DR on CD33- HLA DR+
 HLA DR on CD14+ monocyte
 HLA DR on CD14+ CD16+ monocyte
 HLA DR on CD14+ CD16- monocyte
 HLA DR on CD14- CD16+ monocyte
 HLA DR on CD14- CD16-
 HLA DR on B cell
 FSC-A on myeloid Dendritic Cell
 CX3CR1 on monocyte
 CX3CR1 on CD14+ CD16+ monocyte
 CD62L- Dendritic Cell %Dendritic Cell
 CX3CR1 on CD14- CD16+ monocyte
 CD80 on granulocyte
 CD8+ T cell Absolute Count
 CD8 on Effector Memory CD8+ T cell
 CD64 on monocyte
 CD64 on CD14+ CD16- monocyte
 CD4-CD8- Natural Killer T %lymphocyte
 CD45RA+ CD8+ T cell Absolute Count
 CD4+ CD8dim T cell Absolute Count
 CX3CR1 on CD14+ CD16- monocyte

## Slide 18
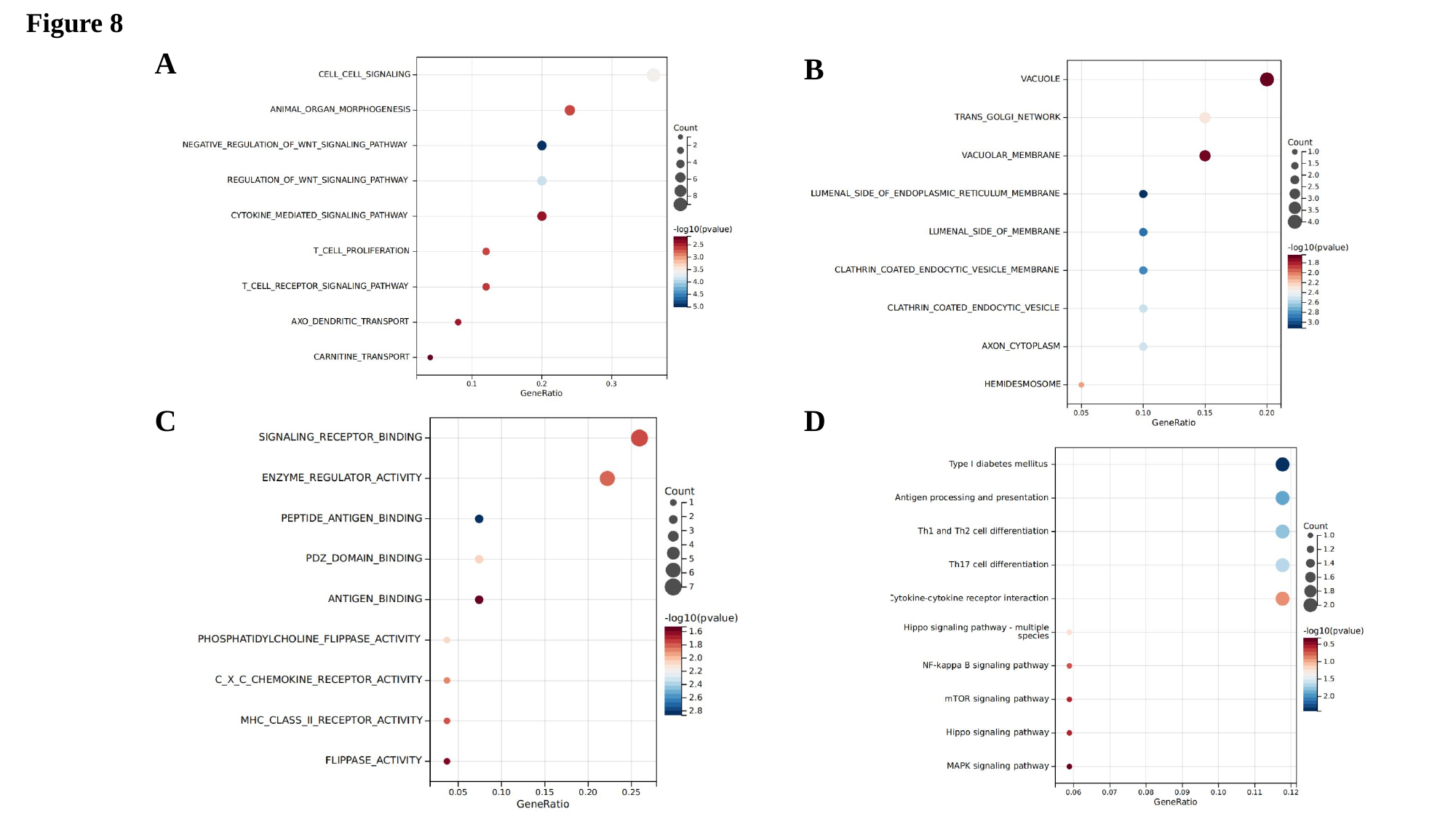

Figure 8
A
B
C
D
