# Supplementary material for: Causal relationship between 731 immune cells and the risk of diabetic nephropathy: a two‑sample bidirectional Mendelian randomization study
Source: Ren Fail. 2024 Aug 1;46(2):2387208. doi: 10.1080/0886022X.2024.2387208 (PMC11299454; doi:10.1080/0886022X.2024.2387208)
Supplement: Change of authorship request.pdf [file IRNF_A_2387208_SM4103.pdf]

Change of authorship request form - Journals

Title of manuscript: Causal relationship between 731 immune cells and the risk of diabetic nephropathy: a two-   sample bidirectional Mendelian randomization study

Journal Name:Renal Failure

Manuscript ID no.:248369336

1. **Current Authorship list, in the order shown on the manuscript. Please indicate the corresponding author with a ***

Current Author list	First name(s)	Family name	Email address
1st author	Siyuan	Song	18260058070@163.com
2nd author	Yuqing	Sun	1623377848@qq.com
3rd author	Jiangyi	Yu*	1401743118@qq.com
4th author			
5th author			
6th author			

Please insert new rows if needed

2. Please provide an explanation for the change in authorship **(including any reasons for removal from authorship list)**.

In the revised paper, Yuqing Sun helped polishing and analyzing the data, so Yuqing Sun was added in the author list.

3. **Proposed new authorship list (including email addresses), in the order it should appear on the manuscript. Please indicate the corresponding author with a ***

New Author list and order	First name(s)	Family name	Email address	Contribution of authors (please see our authorship criteria)
1st author	Siyuan	Song	18260058070@163.com	designed the research
2nd author	Yuqing	Sun	1623377848@qq.com	analyzed the data
3rd author	Jiangyi	Yu*	1401743118@qq.com	designed the

Information Classification: General

				research
4th author				
5th author				
6th author				

Please insert new rows if needed

4. All authors, unchanged, new and removed must sign this declaration.

Signatures can be in the form of docu-sign, or handwritten signatures can be returned as an image file.

Typed names in the signature box WILL NOT be accepted unless accompanied by an additional email confirmation from that co-author agreeing to the new author list and the explanation of changes outlined in Section 2. This email must come from the same email address assigned to that co-author in the above sections.

**Author name (first name, last name)**	**Declaration**	**Signature (if typed please ensure confirmation via email is attached)**	**Date signed**
**Siyuan Song**	**I agree to the new authorship list and contributions shown above in section 3, for the reasons outlined in section 2.**	Siyuan Song	**2024.07.01**
**Yuqing Sun**	**I agree to the new authorship list and contributions shown above in section 3, for the reasons outlined in section 2.**	Yuqing Sun	**2024.07.01**
**Jiangyi Yu**	**I agree to the new authorship list and contributions shown above in section 3, for the reasons outlined in section 2.**	Jiangyi Yu	**2024.07.01**
	**I agree to the new authorship list and contributions shown above in section 3, for the reasons outlined in section 2.**		
	**I agree to the new authorship list and contributions shown above in section 3, for the reasons outlined in section 2.**		
	**I agree to the new**		

Information Classification: General

	**authorship list and contributions shown above in section 3, for the reasons outlined in section 2.**		
